# Impact of comorbidity burden on outcome in patients with cardiogenic shock: A Cardiogenic Shock Working Group analysis

**DOI:** 10.1002/ejhf.70017

**Published:** 2025-09-16

**Authors:** Jonas Sundermeyer, Song Li, Van‐Khue Ton, Rachna Kataria, Elric Zweck, Kevin John, Manreet K. Kanwar, Jaime Hernandez‐Montfort, Shashank S. Sinha, A. Reshad Garan, Jacob Abraham, Vanessa Blumer, Ajar Kochar, Karthikeyan Ranganathan, Gavin W. Hickey, Mohit Pahuja, Scott Lundgren, Sandeep Nathan, Esther Vorovich, Shelley Hall, Wissam Khalife, Andrew Schwartzman, Ju Kim, Oleg Alec Vishnevsky, Justin Fried, Maryjane Farr, Joseph Mishkin, I‐Hui Chang, Onyedika Ilonze, Alexandra Arias, Jun Nakata, Jeffrey Marbach, Hiram Bezerra, Ann Gage, Joyce Wald, Sunu Thomas, Faisal Rahman, Amirali Masoumi, Aasim Afzal, Salman Gohar, Rachel Goodman, Karol D. Walec, Peter Natov, Borui Li, Paavni Sangal, Qiuyue Kong, Peter Zazzali, Neil M. Harwani, Saraschandra Vallabhajosyula, Arvind Bhimaraj, Claudius Mahr, Daniel Burkhoff, Navin K. Kapur

**Affiliations:** ^1^ The Cardiovascular Center, Tufts Medical Center Boston MA USA; ^2^ Department of Cardiology, University Heart and Vascular Center Hamburg University Medical Center Hamburg‐Eppendorf Hamburg Germany; ^3^ Institute for Advanced Cardiac Care, Medical City Healthcare Dallas TX USA; ^4^ Massachusetts General Hospital Boston MA USA; ^5^ Brown University Health Cardiovascular Institute Providence RI USA; ^6^ Department of Cardiology, Pulmonology, and Vascular Medicine, Medical Faculty and University Hospital Düsseldorf Heinrich‐Heine‐University Düsseldorf Germany; ^7^ University of Chicago Chicago IL USA; ^8^ Baylor Scott & White Health Advanced Heart Failure Program Clinic Temple TX USA; ^9^ Inova Heart and Vascular Institute Falls Church VA USA; ^10^ Beth Israel Deaconess Medical Center Boston MA USA; ^11^ Center for Cardiovascular Analytics, Research, & Data Science (CARDS) Providence St. Joseph Research Network Portland OR USA; ^12^ Division of Cardiovascular Medicine Brigham and Women's Hospital Boston MA USA; ^13^ Cardiovascular Institute at Allegheny Health Network Pittsburgh PA USA; ^14^ University of Pittsburgh Medical Center Pittsburgh PA USA; ^15^ University of Oklahoma Health Science Center Oklahoma City OK USA; ^16^ University of Nebraska Medical Center Omaha NE USA; ^17^ Northwestern Medicine Chicago IL USA; ^18^ Baylor University Medical Center Dallas TX USA; ^19^ University of Texas Medical Branch Galveston TX USA; ^20^ Maine Medical Center Portland OR USA; ^21^ Houston Methodist Research Institute Houston TX USA; ^22^ Thomas Jefferson University Hospital Philadelphia PA USA; ^23^ Columbia University Irving Medical Center New York NY USA; ^24^ UT Southwestern Dallas TX USA; ^25^ Atrium Health Sanger Heart and Vascular Institute Charlotte NC USA; ^26^ Banner University Medical Center Phoenix AZ USA; ^27^ Indiana University School of Medicine Indianapolis IN USA; ^28^ Instituto Nacional de Cardiologia Ignacio Chavez Mexico City Mexico; ^29^ Division of Cardiovascular Intensive Care Nippon Medical School Hospital Tokyo Japan; ^30^ Oregon Health State University Portland OR USA; ^31^ Tampa General Hospital Tampa FL USA; ^32^ TriStar Centennial Medical Center Nashville TN USA; ^33^ University of Pennsylvania Philadelphia PA USA; ^34^ University of Washington Medical Center Seattle WA USA; ^35^ Johns Hopkins University Baltimore MD USA; ^36^ Atlantic Health System Morristown NJ USA; ^37^ Baylor Scott and White Plano TX USA; ^38^ Baylor Scott and White Fort Worth TX USA; ^39^ Brown University Health Providence RI USA; ^40^ Houston Methodist Hospital Houston TX USA; ^41^ Cardiovascular Research Foundation New York NY USA

**Keywords:** Cardiogenic shock, Heart failure‐related cardiogenic shock, Acute myocardial infarction‐related cardiogenic shock, Multimorbidity, Comorbidities, Comorbidity burden, Risk stratification

## Abstract

**Aims:**

Comorbidity burden is a major determinant of outcomes. Its prognostic impact on cardiogenic shock (CS) across CS subtypes remains insufficiently characterized. We aimed to characterize the prevalence and distribution of comorbidities in CS, assess their impacts on outcomes, and identify high‐risk comorbidity patterns in all‐cause, acute myocardial infarction‐related (AMI‐CS) and heart failure‐related CS (HF‐CS).

**Methods and results:**

Cardiogenic shock patients from the multicentre Cardiogenic Shock Working Group (CSWG) registry (2020–2024) were analysed. We used adjusted logistic regression models to assess the impact of comorbidities individually, in combination, and as a cumulative burden on in‐hospital mortality. We developed the Comorbidity Risk Index for Cardiogenic Shock (COMRI‐CS) to capture the association between comorbidities and CS mortality. Among 6815 patients (26.5% AMI‐CS, 53.6% HF‐CS), 6087 (89.3%) presented with ≥1 comorbidity, and 4390 (64.4%) with ≥3 comorbidities. In‐hospital mortality increased with comorbidity burden (AMI‐CS: 35.4%, 39.6%, 47.1% with 1–3, 4–6, ≥7 comorbidities, respectively; HF‐CS: 19.6%, 24.9%, 27.5%, respectively). A high comorbidity burden was independently associated with a 51% higher relative mortality risk in AMI‐CS (odds ratio [OR] 1.51, 95% confidence interval [CI] 1.02–2.23, *p* = 0.037), and a more pronounced increase of 122% in HF‐CS (OR 2.22, 95% CI 1.49–3.37, *p* < 0.001). Distinct high‐risk comorbidities and combinations were identified, varying across CS subtypes. With each COMRI‐CS point, in‐hospital mortality increased by ~5.5%.

**Conclusions:**

In this large real‐world CS cohort, comorbidity burden was highly prevalent, varied across subtypes, and was independently associated with mortality. Integrating chronic conditions into early CS risk stratification may enhance clinical decision‐making in CS management.

## Introduction

Cardiogenic shock (CS) is a life‐threatening condition marked by a sudden and severe reduction in cardiac output, leading to critical end‐organ hypoperfusion.^1^, ^2^, ^3^ Despite substantial research efforts in epidemiology, aetiology, phenotyping, shock severity staging, haemodynamic profiling and advances in treatment strategies, short‐term mortality in CS remains exceptionally high, often exceeding 40%.^2^, ^4^, ^5^, ^6^, ^7^, ^8^, ^9^ While early revascularization and temporary micro‐axial flow pump use have demonstrated survival benefits in selected acute myocardial infarction‐related CS (AMI‐CS) cohorts, robust evidence for pharmacological or device‐based treatment options in the broader CS population, particularly in the large proportion of heart failure‐related CS (HF‐CS), remains scarce.^8^, ^10^, ^11^, ^12^, ^13^, ^14^, ^15^, ^16^, ^17^, ^18^

Multimorbidity is a major determinant of prognosis in patients with cardiovascular diseases and represents a growing global challenge.^19^, ^20^ In CS management, physicians must rapidly integrate disease severity, aetiology, haemodynamics, imaging findings, biomarkers, and known chronic pre‐existing conditions, including comorbidity burden, to assess prognosis and guide tailored treatment strategies.^2^ However, the impact of comorbidity burden on CS outcomes remains insufficiently characterized, particularly regarding the role of distinct comorbidities and their cumulative burden, as well as how these effects vary across subtypes. The lack of clinically actionable frameworks identifying which comorbidity patterns exert the greatest prognostic influence, and their implications for CS management, further limits clinical decision‐making and the design of CS trials. Large‐scale CS registries are essential to accurately capture the real‐world prevalence, distribution, and impact of comorbidities, addressing gaps left by randomized controlled trials, which often focus on highly selected CS cohorts with limited generalizability.

This study aimed to assess the prevalence and distribution of comorbidity burden and its impact on clinical outcomes in patients with CS, using a large real‐world cohort from the Cardiogenic Shock Working Group (CSWG) registry. We characterized multimorbidity patterns across CS subtypes (AMI‐CS and HF‐CS), identifying high‐risk comorbidities, their combinations, and thresholds for cumulative burden.

## Methods

### Data source

The CSWG is an academic research consortium comprising centres with CS treatment expertise. This analysis was conducted using the international, multicentre CSWG registry, currently collecting data from CS patients across 36 participating centres. Details on the definition of CS, eligibility criteria, and data entry procedures for the CSWG registry, pre‐defined by principal investigators, have been previously published.^21^, ^22^, ^23^

In summary, this registry includes a standardized set of data elements, including patient demographics, medical history, laboratory, and haemodynamic data at baseline, as well as at specific time points over 72 h, collected prospectively or retrospectively. CS was physician‐adjudicated at each CSWG site and defined as a sustained episode caused by cardiac dysfunction, meeting at least one of the following criteria: systolic blood pressure <90 mmHg for at least 30 min, use of vasoactive agents, cardiac index <2.2 L/min/m^2^ without hypovolaemia, or use of one or more temporary mechanical circulatory support (tMCS) devices for clinically suspected CS. CS patients were treated either conservatively or with tMCS devices, with detailed documentation of the treatment management in the CSWG registry. The CS treatment management was not pre‐defined and was left to the discretion of the treating physicians at the CSWG sites. Data quality was ensured through adjudication, central audits, and resolution of discrepancies by the CSWG principal investigators in collaboration with the submitting sites.

For the final analysis, CSWG data from patients enrolled between 2020 and 2024 were included. CS patients were aetiologically classified as AMI‐CS (defined as a primary diagnosis of non‐ST‐elevation or ST‐elevation myocardial infarction), HF‐CS (including de novo heart failure [HF] or acute‐on‐chronic HF), or other (including post‐cardiotomy shock, graft dysfunction post‐heart transplantation, or unknown CS aetiology). Society for Cardiovascular Angiography & Interventions (SCAI) stages were determined according to the CSWG‐SCAI definition, as previously published.^22^

This analysis adheres to the principles outlined in the Declaration of Helsinki and received approval for patient enrolment from the Institutional Review Boards and local ethics committees of each participating site.

### Definition of study groups

Patients with comorbidities, recorded at the time of index hospitalization by local CSWG investigators, were analysed. A total of 15 major comorbidities were adequately captured in the CSWG registry, including hypertension, diabetes mellitus, chronic kidney disease (CKD), peripheral artery disease (PAD), anaemia, chronic obstructive pulmonary disease (COPD), asthma, liver disease, stroke/transient ischaemic attack (TIA), atrial fibrillation, severe valvular disease, coronary artery disease (CAD), history of known HF, and history of myocardial infarction. For this analysis, cancer was excluded due to the lack of detailed data on its activity, degree of aggressiveness, and its heterogeneous impact on outcome. CS patients were analysed as an overall cohort and stratified into AMI‐CS and HF‐CS. Based on comorbidity burden, they were further categorized into three groups: 1–3 (low), 4–6 (intermediate), and ≥7 comorbidities (high burden).

### Outcome

The primary outcome of this study was in‐hospital mortality. Secondary outcomes included in‐hospital heart replacement therapy, defined as left ventricular assist device (LVAD) implantation or heart transplantation during the index hospitalization, as well as in‐hospital complications (stroke, in‐hospital cardiac arrest, limb ischaemia, acute kidney injury, bleeding requiring surgery, bleeding requiring transfusion, haemolysis).

### Comorbidity Risk Index for Cardiogenic Shock

To derive and internally/externally validate the new Comorbidity Risk Index for Cardiogenic Shock (COMRI‐CS), high‐risk comorbidities associated with in‐hospital outcomes were identified. Additionally, readily available parameters at admission, including age, sex, lactate, creatinine, and CS aetiology, were incorporated based on their prognostic significance and tested in a multivariable logistic regression model. These variables were transformed into a weighted scoring system and stratified into four risk classes (I–IV). Further methodological details regarding COMRI‐CS are provided in online supplementary *Appendix Appendix* *S1*.

### Statistical analyses

Binary variables are presented as absolute numbers and relative frequencies, and comparisons were conducted using Pearson's Chi‐squared test or Fisher's exact test. Continuous variables are shown as the median with interquartile range (IQR) and analysed using the Kruskal–Wallis test.

The prevalence of each distinct comorbidity and various levels of multimorbidity were assessed in the overall cohort and stratified by CS subtype (AMI‐CS vs. HF‐CS).

In‐hospital mortality was assessed in all patients, with no missing data for this outcome. To evaluate the association between comorbidity burden (including distinct comorbidities, combinations, numerical comorbidity levels) and mortality, univariable and multivariable logistic regression models were fitted, adjusted for age and sex.

To evaluate the association between multimorbidity burden and the probability of receiving mechanical circulatory support, vasoactive drug therapy, or mechanical ventilation, logistic regression models were performed. Additionally, a logistic regression model was used to assess whether a higher comorbidity burden at baseline is associated with CS aetiology (HF‐CS vs. AMI‐CS), adjusted for age and sex.

To assess the discriminatory performance of COMRI‐CS, receiver‐operating characteristic curves were calculated. Internal validation was performed using 500 bootstrap resamples. For external validation, identical inclusion/exclusion criteria and statistical analyses were applied on CS patients in the Medical Information Mart for Intensive Care (MIMIC) IV validation cohort, as detailed in online supplementary *Methods S1*.

Odds ratios (OR) and 95% confidence intervals (CI) are presented, a *p*‐value of <0.05 was considered statistically significant. Analyses were performed using R statistical software (version 4.4.1).

## Results

### Study cohort

Among 11 244 patients in the CSWG registry, 6815 patients met the inclusion criteria for this study (*Graphical Abstract*). The median age was 62 years (IQR 52–70), and 70.3% were male. Among these patients, 3652 (53.6%) presented with HF‐CS and 1809 (26.5%) with AMI‐CS. Baseline lactate was 2.3 mmol/L (IQR 1.4–4.4), creatinine 1.4 (1.1–1.9) mg/dl, and left ventricular ejection fraction was 22% (IQR 15–35). A total of 862 (12.8%) patients had prior cardiac arrest.

Baseline characteristics for the overall cohort and stratified by low (1–3 comorbidities), intermediate (4–6 comorbidities), and high (≥7 comorbidities) comorbidity burden are detailed in *Table* *1*. Patients with a higher comorbidity burden were older (60 vs. 64 vs. 66 years) and had higher baseline creatinine levels (1.3 vs. 1.5 vs. 1.7 mg/dl). Blood pressure profiles, pulmonary capillary wedge pressure, and cardiac output did not differ significantly across groups. Patients with a higher comorbidity burden were more likely to present with lower baseline SCAI shock stages. Additionally, the likelihood of out‐of‐hospital cardiac arrest decreased with increasing comorbidity burden (14.7% vs. 9.3% vs. 4.5%). Characteristics stratified by HF‐CS versus AMI‐CS are detailed in online supplementary *Table Appendix* *S1*.

**Table 1:** Characteristics for the overall cohort and stratified by comorbidity burden

	Overall (*n* = 6815)	Comorbidities				*p*‐value
		0 (*n* = 728)	1–3 (*n* = 2645)	4–6 (*n* = 2456)	≥7 (*n* = 986)	
**Demographics**						
Male sex	4791/6811 (70.3)	498/727 (68.5)	1753/2644 (66.3)	1814/2455 (73.9)	726/985 (73.7)	<0.001
Age (years)	62 (52–70)	55 (43–66)	60 (48–68)	64 (55–72)	66 (60–73)	<0.001
Race						
Black	1349/6815 (19.8)	73/728 (10)	473/2645 (17.9)	587/2456 (23.9)	216/986 (21.9)	<0.001
White	4393/6815 (64.5)	509/728 (69.9)	1674/2645 (63.3)	1547/2456 (63)	663/986 (67.2)	<0.001
Asian	275/6815 (4)	38/728 (5.2)	132/2645 (5)	78/2456 (3.2)	27/986 (2.7)	<0.001
Other	264/6815 (3.9)	39/728 (5.4)	123/2645 (4.7)	75/2456 (3.1)	27/986 (2.7)	<0.001
Body mass index (kg/m^2^)	28 (24.2–32.7)	26.7 (23.6–31.2)	27.8 (24.1–32.4)	28.3 (24.6–33)	28.9 (24.5–33.7)	<0.001
**Medical history**						
Hypertension	4403/6774 (65)	0/706 (0)	1438/2630 (54.7)	2022/2452 (82.5)	943/986 (95.6)	<0.001
Diabetes mellitus	2742/6785 (40.4)	0/712 (0)	730/2634 (27.7)	1267/2453 (51.7)	745/986 (75.6)	<0.001
Atrial fibrillation/flutter	2155/6778 (31.8)	0/707 (0)	387/2632 (14.7)	1097/2453 (44.7)	671/986 (68.1)	<0.001
Chronic kidney disease	2014/6780 (29.7)	0/710 (0)	225/2631 (8.6)	1040/2455 (42.4)	749/984 (76.1)	<0.001
Peripheral vascular disease	629/6777 (9.3)	0/708 (0)	79/2631 (3)	237/2453 (9.7)	313/985 (31.8)	<0.001
COPD	904/6782 (13.3)	0/711 (0)	152/2634 (5.8)	391/2452 (15.9)	361/985 (36.6)	<0.001
Asthma	550/6524 (8.4)	0/645 (0)	176/2533 (6.9)	203/2386 (8.5)	171/960 (17.8)	<0.001
Cancer	0/6617 (0)	0/648 (0)	0/2560 (0)	0/2431 (0)	0/978 (0)	>0.9
Liver disease	310/6585 (4.7)	0/646 (0)	57/2547 (2.2)	144/2419 (6)	109/973 (11.2)	<0.001
Anaemia	1155/6528 (17.7)	0/643 (0)	172/2525 (6.8)	464/2399 (19.3)	519/961 (54)	<0.001
History of stroke/TIA	869/6782 (12.8)	0/711 (0)	140 / 2634 (5.3)	385/2452 (15.7)	344/985 (34.9)	<0.001
Severe valve disease	1377/6777 (20.3)	0/709 (0)	245/2633 (9.3)	662/2450 (27)	470/985 (47.7)	<0.001
Prior CAD	2714/6464 (42)	0/632 (0)	443/2461 (18)	1410/2393 (58.9)	861/978 (88)	<0.001
History of HF	3992/6773 (58.9)	0/706 (0)	1045/2626 (39.8)	2011/2455 (81.9)	936/986 (94.9)	<0.001
History of MI	1466/6775 (21.6)	0/707 (0)	171/2630 (6.5)	710/2454 (28.9)	585/984 (59.5)	<0.001
No. of comorbidities	4 (2–5)	0 (0–0)	2 (1–3)	5 (4–6)	8 (7–8)	<0.001
**Aetiology of shock**						
AMI‐CS	1809/6815 (26.5)	310/728 (42.6)	895/2645 (33.8)	447/2456 (18.2)	157/986 (15.9)	<0.001
HF‐CS	3652/6815 (53.6)	226/728 (31)	1183/2645 (44.7)	1555/2456 (63.3)	688/986 (69.8)	<0.001
OHCA	862/6752 (12.8)	206/722 (28.5)	385/2624 (14.7)	227/2429 (9.3)	44/977 (4.5)	<0.001
**Laboratory values on admission**						
Lactate (mEq/L)	2.3 (1.4–4.4)	3.5 (1.8–8.6)	2.5 (1.6–5)	2 (1.3–3.6)	1.9 (1.3–3.2)	<0.001
pH	7.4 (7.3–7.4)	7.3 (7.2–7.4)	7.4 (7.3–7.4)	7.4 (7.3–7.4)	7.4 (7.3–7.4)	<0.001
ALT (IU/L)	34 (19–74)	59.5 (32–110.5)	42.5 (23–88)	29 (17–59)	25 (15–46)	<0.001
AST (IU/L)	44 (26–101)	84.5 (39–193)	55 (29–134)	38 (23–77)	32 (21–61)	<0.001
Serum creatinine (mg/dl)	1.4 (1.1–1.9)	1.2 (0.9–1.6)	1.3 (1–1.7)	1.5 (1.1–2.1)	1.7 (1.3–2.3)	<0.001
Blood urea nitrogen (mg/dl)	27 (18–41)	20 (14–29)	23 (16–35)	30 (21–45)	37 (25–52)	<0.001
Sodium (mEq/L)	137 (133–139)	138 (135–140)	137 (133.4–139)	136 (133–139)	136 (133–139)	<0.001
Potassium (mEq/L)	4.1 (3.7–4.6)	4 (3.7–4.5)	4.1 (3.7–4.5)	4.2 (3.8–4.6)	4.2 (3.8–4.7)	<0.001
HCO_3_ (mEq/L)	22 (19–25)	21 (17–24)	21.3 (18.6–25)	23 (20–26)	23 (20–26)	<0.001
Total bilirubin (mg/dl)	0.9 (0.6–1.6)	0.8 (0.5–1.3)	0.9 (0.5–1.6)	1 (0.6–1.6)	0.9 (0.6–1.5)	<0.001
Haemoglobin (g/dl)	12.3 (10.4–14)	13.1 (11.1–14.7)	12.6 (10.9–14.2)	12.1 (10.3–13.7)	11.3 (9.6–13.1)	<0.001
**Echocardiography at baseline**						
LVEF (%)	22 (15–35)	20.5 (15–33)	22.5 (15–37)	21.1 (15–32.5)	22.5 (17–33)	0.040
**Haemodynamics**						
SBP (mmHg)	109 (97–126)	109 (94–126)	109 (96–128)	110 (97–125)	110 (98–127)	0.3
DBP (mmHg)	71 (61–82)	72 (60–84)	72 (61–83)	71 (61–82)	70 (60–80)	0.002
Heart rate (bpm)	91 (77–108)	99 (81.5–117)	95 (79–110)	89 (75–104)	88 (75–103)	<0.001
MAP (mmHg)	83 (73–93)	82 (71.5–93)	83 (73–95)	83 (74–93)	82 (74–91)	0.30
Central venous pressure (mmHg)	12 (8–17)	12 (8–16)	12 (8–17)	12 (8–17)	12 (8–18)	>0.9
Right atrial pressure (mmHg)	12 (9–17)	12.5 (8.5–16)	12 (9–17)	12 (9–16)	13 (9–19)	0.7
Pulmonary artery systolic pressure (mmHg)	42 (32–52)	38 (29–47)	39 (30–50)	45 (36–56)	51 (41–60)	<0.001
Pulmonary artery diastolic pressure (mmHg)	23 (18–29)	21.5 (16–27)	22 (17–28)	23 (18–30)	27 (21–30)	<0.001
Pulmonary capillary wedge pressure (mmHg)	22 (16–27.5)	18 (14–26)	23 (17–27)	21 (16–28)	24 (17.5–30.5)	0.2
Cardiac output (L/min)	4 (3.1–5)	3.5 (2.8–4.8)	3.9 (3.2–5)	4.1 (3.1–5.1)	4.4 (3.4–4.8)	0.3
**SCAI shock stage at baseline**						
SCAI B	987/6815 (14.5)	81/728 (11.1)	381/2645 (14.4)	366/2456 (14.9)	159/986 (16.1)	0.028
SCAI C	1101/6815 (16.2)	86/728 (11.8)	380/2645 (14.4)	453/2456 (18.4)	182/986 (18.5)	<0.001
SCAI D	966/6815 (14.2)	126/728 (17.3)	436/2645 (16.5)	311/2456 (12.7)	93/986 (9.4)	<0.001
SCAI E	1056/6815 (15.5)	213/728 (29.3)	484/2645 (18.3)	277/2456 (11.3)	82/986 (8.3)	<0.001
**Mechanical circulatory support**						
VA‐ECMO	1066/6815 (15.6)	165/728 (22.7)	512/2645 (19.4)	283/2456 (11.5)	106/986 (10.8)	<0.001
Impella CP	619/6815 (9.1)	107/728 (14.7)	287/2645 (10.9)	176/2456 (7.2)	49/986 (5)	<0.001
Impella 2.5	5/6815 (0.1)	1/728 (0.1)	1/2645 (0)	3/2456 (0.1)	0/986 (0)	0.4
Impella 5.0	21/6815 (0.3)	4/728 (0.5)	7/2645 (0.3)	9/2456 (0.4)	1/986 (0.1)	0.4
Impella 5.5	900/6815 (13.2)	76/728 (10.4)	353/2645 (13.3)	344/2456 (14)	127/986 (12.9)	0.10
IABP	1494/6815 (21.9)	117/728 (16.1)	568/2645 (21.5)	564/2456 (23)	245/986 (24.8)	<0.001
**Other treatments**						
No. of vasoactive drugs max						
0	273/3794 (7.2)	22/402 (5.5)	100/1473 (6.8)	119/1411 (8.4)	32/508 (6.3)	
1	1237.0/3794.0 (32.6)	95/402 (23.6)	453/1473 (30.8)	504/1411 (35.7)	185/508 (36.4)	
2	982/3794 (25.9)	126/402 (31.3)	395/1473 (26.8)	340/1411 (24.1)	121/508 (23.8)	
3	770/3794 (20.3)	95/402 (23.6)	312/1473 (21.2)	266/1411 (18.9)	97/508 (19.1)	
4	423/3794 (11.1)	52/402 (12.9)	170/1473 (11.5)	137/1411 (9.7)	64/508 (12.6)	
5	88/3794 (2.3)	8/402 (2.0)	36/1473 (2.4)	37/1411 (2.6)	7/508 (1.4)	
Mechanical ventilation	4365/6779 (64.4)	543/727 (74.7)	1817/2630 (69.1)	1449/2440 (59.4)	556/982 (56.6)	<0.001
RRT	1568/6789 (23.1)	153/725 (21.1)	549/2634 (20.8)	593/2450 (24.2)	273/980 (27.9)	<0.001

Binary variables are presented as absolute numbers and relative frequencies, and comparisons were conducted using Pearson's Chi‐squared test or Fisher's exact test. Continuous variables are shown as the median with interquartile range and analysed using the Kruskal–Wallis test.

ALT, alanine aminotransferase; AMI‐CS, acute myocardial infarction‐related cardiogenic shock; AST, aspartate aminotransferase; CAD, coronary artery disease; COPD, chronic obstructive pulmonary disease; HCO_3_, sodium bicarbonate; HF, heart failure; HF‐CS, heart failure‐related cardiogenic shock; IABP, intra‐aortic balloon pump; LVEF, left ventricular ejection fraction; MAP, mean arterial pressure; MI, myocardial infarction; OHCA, out‐of‐hospital cardiac arrest; RRT, renal replacement therapy; SBP, systolic blood pressure; SCAI, Society for Cardiovascular Angiography & Interventions; TIA, transient ischaemic attack; VA‐ECMO, veno‐arterial extracorporeal membrane oxygenation.

### Prevalence of comorbidities in cardiogenic shock

**Figure 1:**
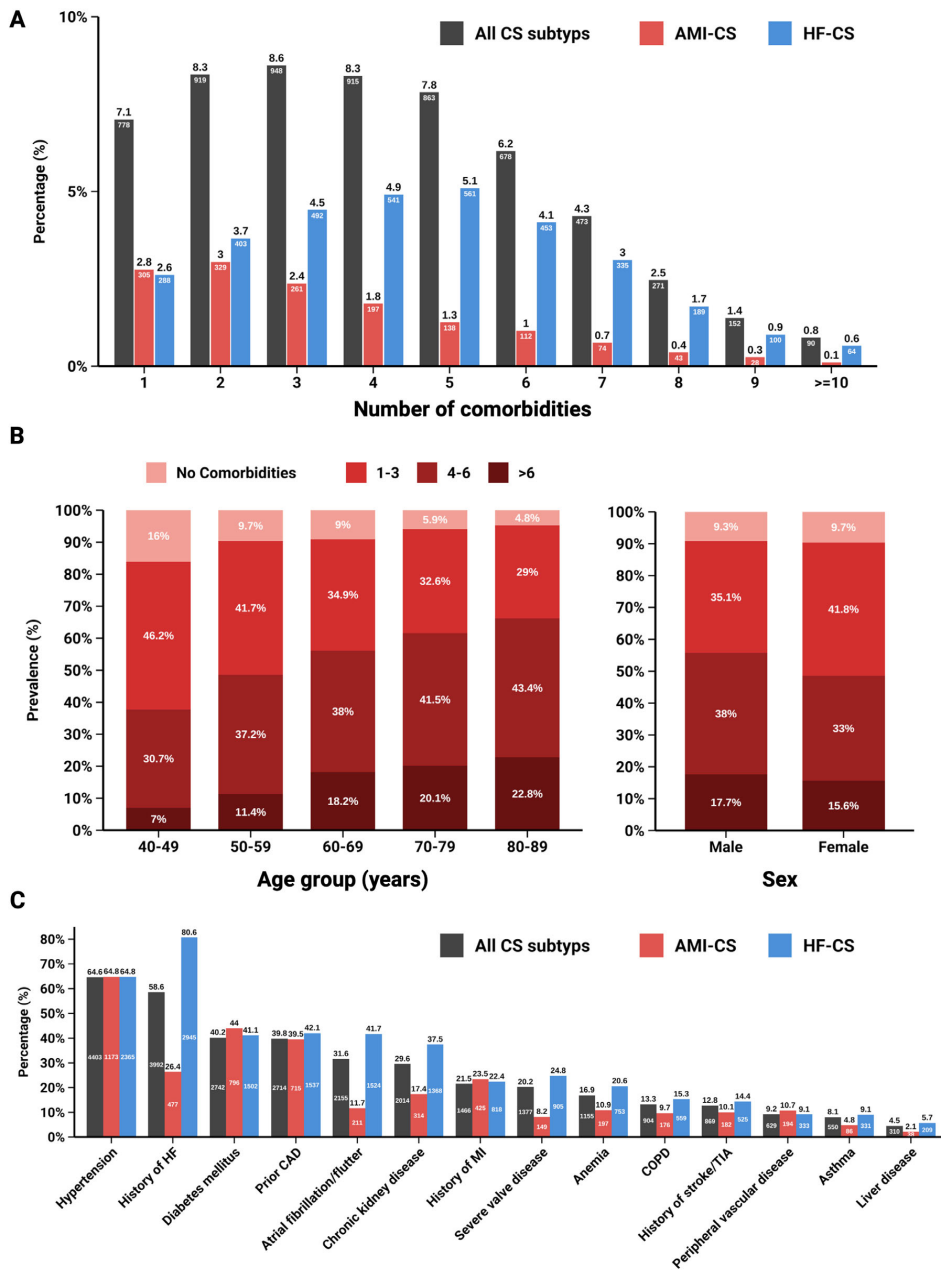
Prevalence of comorbidity burden. Prevalence of multimorbidity by number of comorbidities (*A*), across the age and sex spectrum (*B*), and distinct comorbidities (*C*), shown for all‐cause cardiogenic shock (CS), acute myocardial infarction‐related CS (AMI‐CS) and heart failure‐related CS (HF‐CS). CAD, coronary artery disease; COPD, chronic obstructive pulmonary disease; HF, heart failure; MI, myocardial infarction; TIA, transient ischaemic attack.

In all‐cause CS, 89.3% of patients presented with ≥1 comorbidity, and 64.4% with ≥3. The burden of comorbidity was notably higher in HF‐CS compared to AMI‐CS, with 74.9% vs. 47.8% of patients having ≥3, and 31.2% vs. 14.9% having ≥6 comorbidities (online supplementary *Table* *S2*). The absolute number of comorbidities across the entire cohort, stratified by HF‐CS and AMI‐CS, is illustrated in *Figure* *1A*. A higher comorbidity burden was significantly associated with HF‐CS (adjusted OR [aOR] 1.91, 95% CI 1.57–2.33 for low; 5.93, 95% CI 4.81–7.32 for intermediate; and 8.32, 95% CI 6.47–10.75 for high comorbidity burden; *p* < 0.001; online supplementary *Figure Appendix* *S1*). Age‐stratified analysis showed a progressive increase in comorbidity burden with advancing age, sex‐based differences were not observed (*Figure* *1B*).

The distribution of distinct comorbidities across CS subtypes is illustrated in *Figure* *1C*. Hypertension was the most prevalent comorbidity (64.6%), followed by a history of HF (58.6%), diabetes (40.2%), and CAD (39.8%). Compared to AMI‐CS, HF‐CS patients had a markedly higher prevalence of HF history (80.6% vs. 26.4%), reflecting a high prevalence of acute‐on‐chronic HF‐CS at admission, as well as higher rates of atrial fibrillation (41.7% vs. 11.7%) and CKD (37.5% vs. 17.4%).

### Comorbidity burden associated with mortality and complications

**Figure 2:**
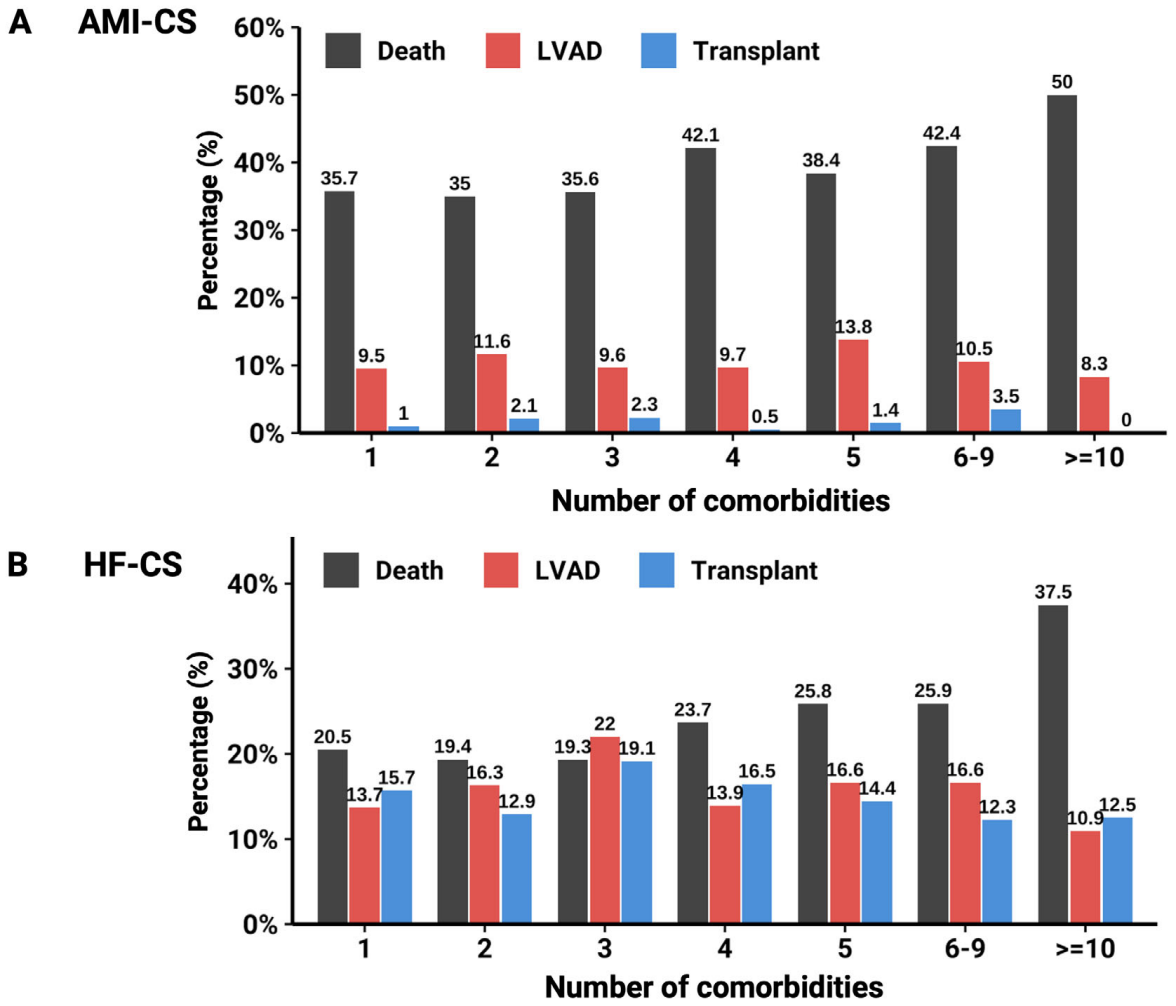
Clinical outcome across different comorbidity levels in acute myocardial infarction‐related cardiogenic shock (AMI‐CS) and heart failure‐related cardiogenic shock (HF‐CS). Crude in‐hospital outcome stratified by number of comorbidities in AMI‐CS (*A*) and HF‐CS (*B*). LVAD, left ventricular assist device.

In the overall cohort, 1991 (29.2%) patients died in‐hospital. Crude in‐hospital mortality was 23.0% in HF‐CS and 37.8% in AMI‐CS (online supplementary *Figure* *S2*). In‐hospital mortality progressively increased with higher comorbidity burden, as well as across distinct multimorbidity strata (*Figure* *2>A, B*) within CS subgroups. Specifically, mortality escalated from 35.4% to 39.6% to 47.1% in AMI‐CS (1–3, 4–6, ≥7 comorbidities, respectively) and 19.6% to 24.9% to 27.5% in HF‐CS (*Graphical Abstract*).

**Figure 3:**
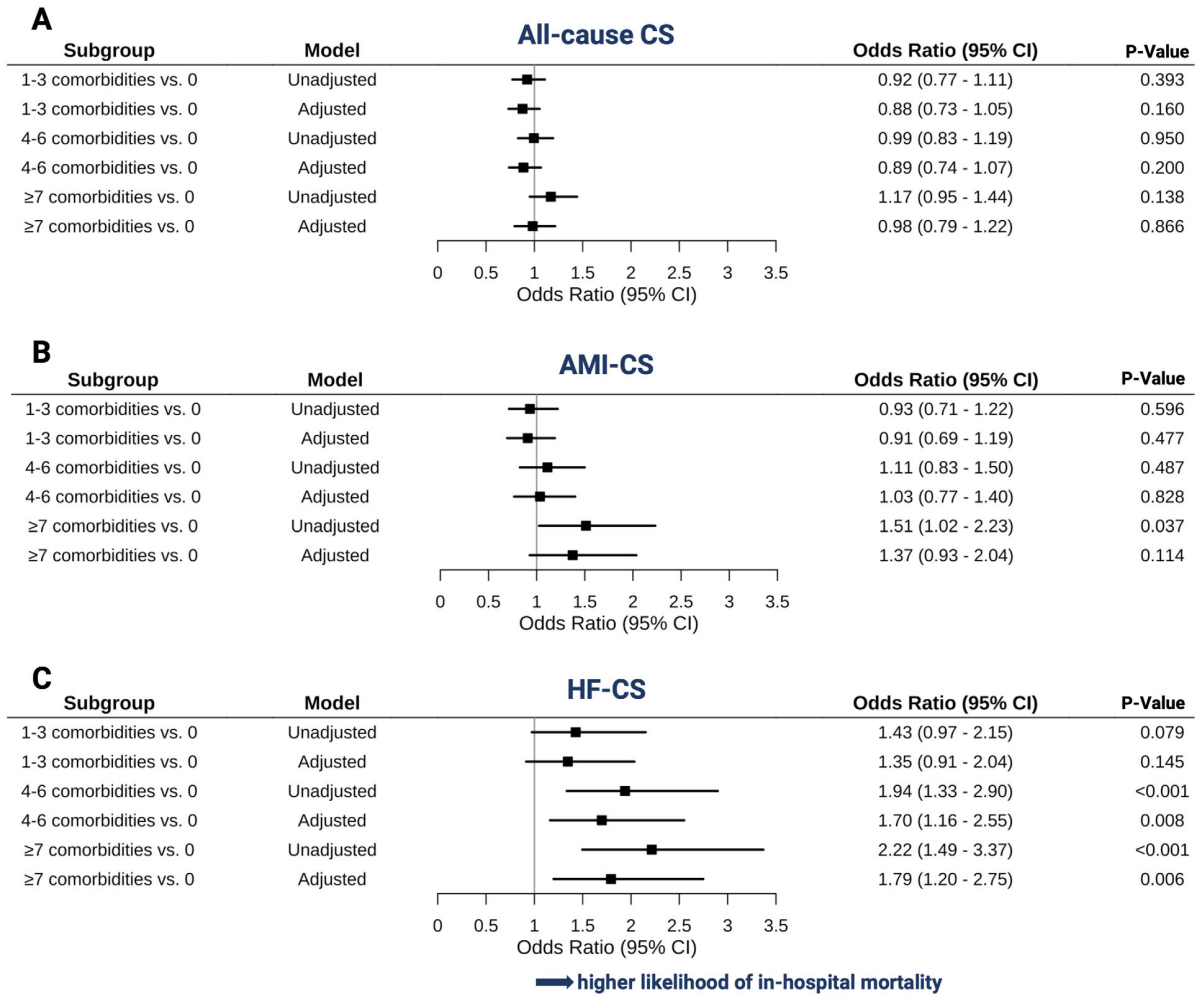
Association between multimorbidity level and in‐hospital mortality. Odds ratios for in‐hospital mortality, calculated by multivariable logistic regression models in the overall cohort (*A*), in acute myocardial infarction‐related cardiogenic shock (AMI‐CS) (*B*), and in heart failure‐related cardiogenic shock (HF‐CS) (*C*), adjusted by age and sex. CI, confidence interval.

In all‐cause CS, a high comorbidity burden was not associated with a higher mortality risk (*Figure* *3A*). In AMI‐CS subjects, a high comorbidity burden was independently associated with a 51% higher relative mortality risk in the unadjusted models (OR 1.51, 95% CI 1.02–2.23, *p* = 0.037; *Figure* *3B*). In HF‐CS subjects, a high comorbidity burden was independently associated with a 122% higher relative mortality risk (OR 2.22, 95% CI 1.49–3.37, *p* < 0.001; *Figure* *3C*). A non‐linear association between comorbidity burden and in‐hospital mortality was observed, with a progressive increase beyond approximately seven comorbidities (online supplementary *Figure* *S3*).

**Figure 4:**
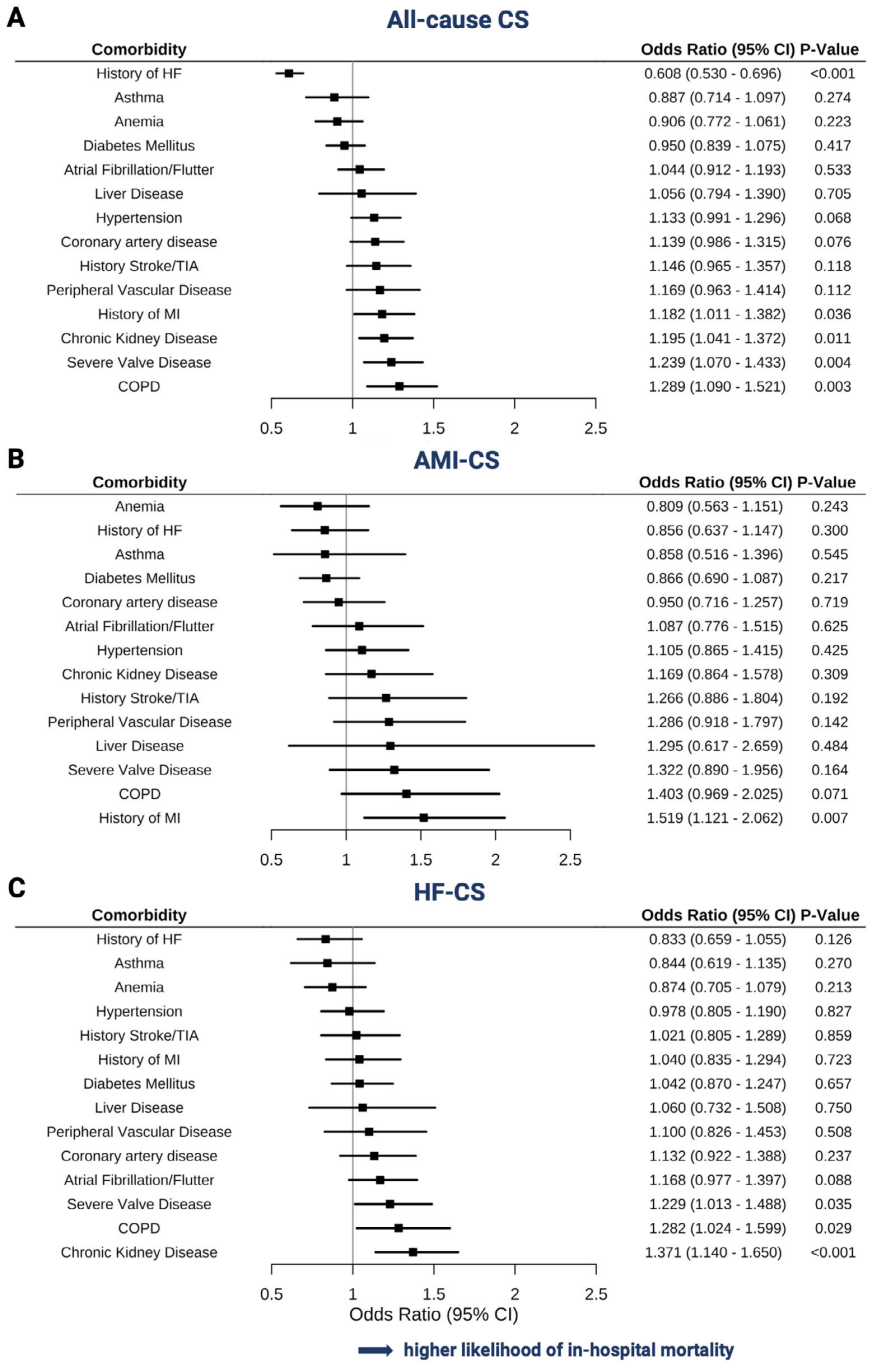
Association between distinct comorbidities and in‐hospital mortality. Odds ratios for in‐hospital mortality, calculated by multivariable logistic regression models in all‐cause cardiogenic shock (CS) (*A*), in acute myocardial infarction‐related CS (AMI‐CS) (*B*), and in heart failure‐related CS (HF‐CS) (*C*), adjusted by age and sex. CI, confidence interval; COPD, chronic obstructive pulmonary disease; HF, heart failure; MI, myocardial infarction; TIA, transient ischaemic attack.

Across distinct comorbidities, COPD was the strongest overall predictor of in‐hospital mortality in all‐cause CS (aOR 1.29, 95% CI 1.09–1.52, *p* = 0.003; *Figure* *4A*). In AMI‐CS, a history of myocardial infarction conferred the highest mortality risk (aOR 1.52, 95% CI 1.12–2.06, *p* = 0.007; *Figure* *4B*), whereas in HF‐CS, CKD was the strongest predictor (aOR 1.37, 95% CI 1.14–1.65, *p* < 0.001; *Figure* *4C*). The unadjusted association between distinct comorbidities and in‐hospital mortality across CS subtypes is illustrated in online supplementary*Figure* *S4*.

**Figure 5:**
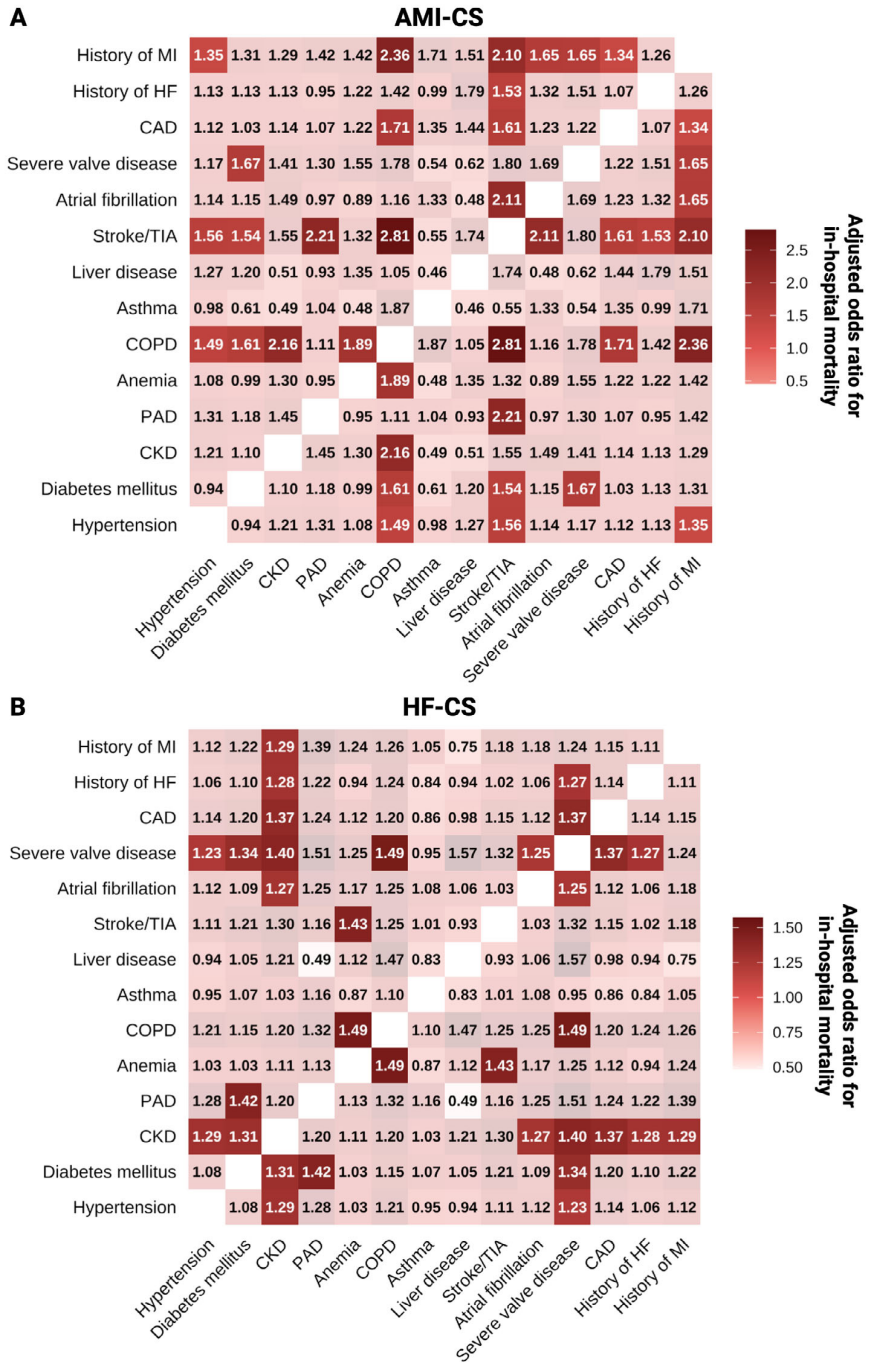
Association between distinct combinations of comorbidities and in‐hospital mortality. Association between distinct comorbidity combinations and mortality, calculated using multivariable logistic regression models in acute myocardial infarction‐related cardiogenic shock (AMI‐CS) (*A*) and heart failure‐related cardiogenic shock (HF‐CS) (*B*), adjusted for age, sex, lactate, creatinine, and out‐of‐hospital cardiac arrest. Statistically significant results (*p* < 0.05) are highlighted in colour. CAD, coronary artery disease; CKD, chronic kidney disease; COPD, chronic obstructive pulmonary disease; HF, heart failure; MI, myocardial infarction; PAD, peripheral artery disease; TIA, transient ischaemic attack.

Distinct high‐risk comorbidity combinations associated with in‐hospital mortality varied across CS subtypes. In all‐cause CS, a high risk was observed for the combinations of COPD plus severe valve disease (aOR 1.45, 95% CI 1.11–1.89, *p* = 0.006) and COPD plus stroke/TIA (aOR 1.44, 95% CI 1.04–1.99, *p* = 0.027; online supplementary *Figure* *S5*). In AMI‐CS, COPD plus stroke/TIA (aOR 2.81, 95% CI 1.39–5.95, *p* = 0.005; *Figure* *5A*) conferred a high mortality risk. In HF‐CS, a high risk was observed for COPD plus severe valve disease (aOR 1.49, 95% CI 1.05–2.08, *p* = 0.023; *Figure* *5B*). Whereas in AMI‐CS, high‐risk combinations frequently included COPD and stroke/TIA, in HF‐CS, CKD was predominantly involved in high‐risk combinations.

The prevalence of in‐hospital complications varied by comorbidity burden, with acute kidney injury showing a rising trend as comorbidity burden increased (online supplementary*Figure* *S6*). A higher comorbidity burden remained independently associated with an increased risk of acute kidney injury in both AMI‐CS (OR for high comorbidity burden: 1.72, 95% CI 1.21–2.47, *p* = 0.003) and HF‐CS (OR for high comorbidity burden: 1.61, 95% CI 1.32–1.96, *p* < 0.001; online supplementary *Figure* *S7*).

### Association between comorbidity burden and selected treatments

Comorbidity burden was not associated with the use of vasoactive agents or the number of drugs used. However, a high comorbidity burden was linked to a 27% lower likelihood of receiving tMCS (OR 0.73, 95% CI 0.63–0.85, *p* < 0.001) compared with a low comorbidity burden. Among patients receiving tMCS, those with a higher comorbidity burden were also less likely to receive multiple devices (OR 0.74, 95% CI 0.61–0.90, *p* = 0.003). Moreover, a high comorbidity burden was associated with a 42% lower probability of receiving mechanical ventilation (OR 0.58, 95% CI 0.50–0.68, *p* < 0.001) and a 34% lower likelihood of prolonged ventilation duration (OR 0.66, 95% CI 0.54–0.81, *p* < 0.001). These associations were less pronounced after stratification by AMI‐CS and HF‐CS (online supplementary *Figure* *S8*).

### Comorbidity Risk Index for Cardiogenic Shock

**Figure 6:**
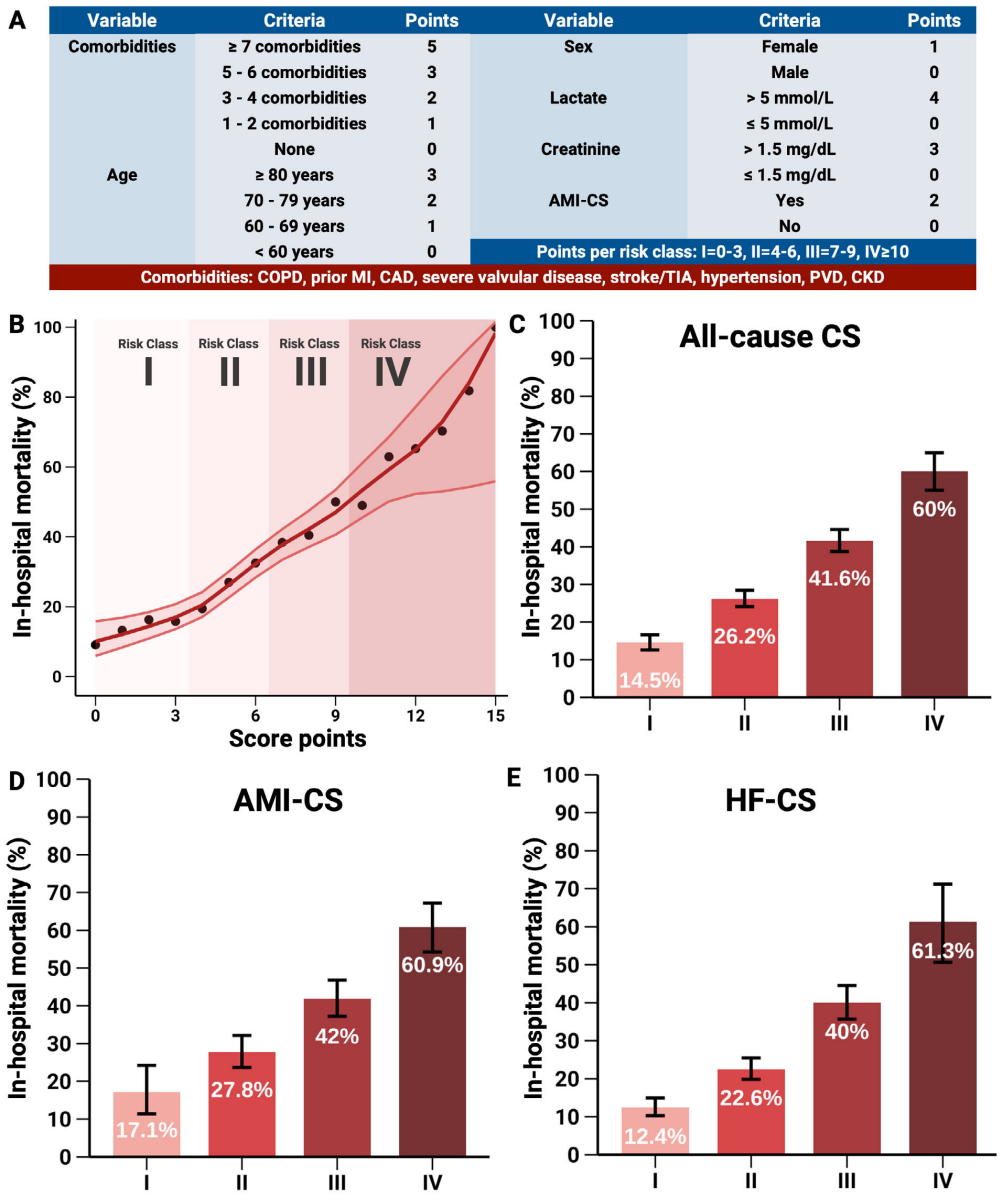
Comorbidity Risk Index for Cardiogenic Shock (COMRI‐CS). COMRI‐CS based on high‐risk comorbidities, age, sex, lactate, creatinine, and acute myocardial infarction‐related cardiogenic shock (AMI‐CS) status, with risk stratification into four risk classes (*A*). With each additional COMRI‐CS point, in‐hospital mortality increased by approximately 5.5% (95% confidence intervals presented per point, *B*). In‐hospital mortality increased progressively across risk classes for all‐cause cardiogenic shock (CS) (*C*), AMI‐CS (*D*), and heart failure‐related CS (HF‐CS) (*E*). CAD, coronary artery disease; CKD, chronic kidney disease; COPD, chronic obstructive pulmonary disease; MI, myocardial infarction; PVD, peripheral vascular disease; TIA, transient ischaemic attack.

Each additional COMRI‐CS score point was associated with a ~5.5% increase of in‐hospital mortality (*Figure* *6A, B*). Stratification into four risk classes (I–IV) showed a clear gradient of in‐hospital mortality among CS subgroups (*Figure* *6C–E*: 14.5%, 26.2%, 41.6%, and 60.0% for all‐cause CS (area under the curve [AUC]: 0.691, 95% CI 0.674–0.708); 17.1%, 27.8%, 42.0%, and 60.9% for AMI‐CS (AUC: 0.680, 95% CI 0.649–0.710); 12.4%, 22.6%, 40%, and 61.3% for HF‐CS (AUC: 0.693, 95% CI 0.668–0.718). The number of patients across risk classes and AUC bootstrap distribution are provided in online supplementary *Figure* *S9*. In the validation cohort, risk stratification across classes I–IV remained consistent, with in‐hospital mortality rates of 23.5%, 34.9%, 50.7%, and 68.9% (online supplementary *Figure* *S10*).

## Discussion

In this large, multicentre, contemporary cohort of 6815 CS patients, comorbidity burden was highly prevalent, with nearly 75% of patients presenting with three or more comorbidities. HF‐CS patients exhibited a higher comorbidity burden compared to AMI‐CS. In patients with HF‐CS, a high comorbidity burden was associated with a 79% higher relative risk of in‐hospital mortality. Non‐linear modelling revealed a critical threshold at approximately seven comorbidities, beyond which mortality risk increased disproportionately. Distinct high‐risk comorbidities and combinations were identified, varying across CS subtypes. With COMRI‐CS, the present report also provides a scoring system that incorporates high‐risk comorbidities beyond standard demographic and CS severity measures. COMRI‐CS demonstrated effective risk stratification across its defined risk classes, with each score point increasing in‐hospital mortality by approximately 5.5%. Moreover, treatment disparities were evident, as patients with a higher comorbidity burden were less likely to receive tMCS and mechanical ventilation.

### Prevalence of comorbidities in cardiogenic shock

In patients with CS, assessing comorbidity burden is recognized as a key determinant of risk prediction.^2^ While recent findings emphasize its high prevalence, data across distinct CS subtypes in real‐world cohorts remain limited.^24^ A more detailed understanding of comorbidity burden, including subtype‐specific patterns and prognostic implications is crucial for developing clinically actionable frameworks that enable high‐risk subpopulations identification to guide clinical decision‐making, and was therefore further investigated in this study.

In this large, multicentre study, multimorbidity was highly prevalent, with nearly 90% of CS patients presenting with at least one comorbidity, and 75% with three or more comorbidities. The comorbidity burden was significantly higher in HF‐CS than in AMI‐CS, with ≥3 comorbidities in 75% versus 48% of patients and ≥6 comorbidities in 31% versus 15%, respectively. These findings are consistent with prior studies, demonstrating that HF‐CS patients exhibit a higher chronic disease burden.^8^, ^25^, ^26^ This likely reflects the cumulative impact of longstanding HF, where prolonged haemodynamic stress and systemic neurohormonal activation increase susceptibility to further end‐organ damage, promoting the accumulation of comorbidities.^26^ Recent CS phenotyping analyses have identified distinct subgroups, including non‐congested, cardiorenal, and cardiometabolic phenotypes, with the cardiorenal phenotype carrying the highest comorbidity burden, underscoring the complex interplay between preexisting conditions and clinical CS course.^9^

The most common comorbidities in AMI‐CS were hypertension (65%), diabetes (44%), prior CAD (40%), and known HF (26%), with slightly higher prevalence rates compared to a recent multicentre retrospective Canadian study on AMI‐CS.^24^ In contrast, HF‐CS patients had a markedly higher burden of known HF (81%) and atrial fibrillation (42% vs. 12%) and CKD (42% vs. 21%), compared to AMI‐CS, while hypertension, diabetes, and prior CAD were similarly prevalent in both subtypes. Comparable contemporary HF‐CS cohorts are limited, often representing selected sub‐cohorts and capturing only a subset of comorbidities, making direct comparisons challenging.^8^, ^25^

### Comorbidity burden associated with outcome

While early CS evaluation and prognostication, particularly within the first 24 h of CS, play a pivotal role in outcome prediction, CS aetiologies (AMI vs. HF‐CS, including de novo vs. acute‐on‐chronic HF‐CS) and distinct clinical and haemodynamic phenotypes significantly impact prognosis, posing challenges in the diagnosis and management of highly heterogeneous CS populations.^1^, ^21^, ^23^, ^27^ The third axis includes distinct risk modifiers, integrating the presence and reversibility of organ failure, cardiac arrest with evidence of anoxic of brain injury, additional factors such as age and the broad spectrum of comorbidities.^2^ While some risk modifiers may be directly therapeutically addressable, preexisting comorbidities represent a more static variable, not immediately modifiable, yet plays a critical role in early risk prediction and may influence candidacy for advanced invasive therapeutic interventions.

In this study, a higher comorbidity burden was associated with a stepwise increase in mortality, with a particularly high relative impact in HF‐CS compared to AMI‐CS after adjustment for age and sex. Furthermore, a non‐linear relationship between comorbidity burden and in‐hospital mortality was observed, with a steep increase beyond a threshold of approximately seven comorbidities. These findings align with previous studies demonstrating the quantitative impact of multimorbidity on mortality in large HF cohorts, yet this association has not been previously shown in CS.^26^, ^28^, ^29^ In a recent study providing a comprehensive characterization of non‐cardiac comorbidities in acute HF, a greater number of non‐cardiac comorbidities at admission was similarly associated with longer HF duration and a stepwise increase in mortality risk with increasing comorbidity burden.^26^ Exceeding a critical threshold may amplify mortality risk due to a limited physiological reserve, increased end‐organ vulnerability, and a reduced response to CS therapies, emphasizing the need for a cumulative multimorbidity‐aware risk stratification, particularly in the predisposed subgroup of acute‐on‐chronic HF‐CS.^8^, ^14^

As expected, older patients presented with a higher comorbidity burden. Importantly, while age has been described as a marker of comorbidities and is linked to increased mortality, this study demonstrated that the impact of comorbidity burden on mortality remained significant independent of age, underscoring its prognostic relevance beyond ageing alone.^30^ Novel imaging studies assessing biological organ age instead of chronological age are needed to provide better risk stratification among CS patients with chronic illnesses.

Certain comorbidity combinations and patterns may adversely affect clinical outcomes, though this relationship remains incompletely characterized in the context of CS.^31^ Our findings indicate that COPD was the strongest overall predictor of in‐hospital mortality in all‐cause, whereas a history of myocardial infarction and CKD conferred the highest mortality risk in AMI‐CS and HF‐CS. Despite notable variations across CS subtypes, distinct high‐risk comorbidity combinations associated with mortality frequently involved COPD. This might be driven by chronic inflammation, impaired oxygenation, right ventricular dysfunction, and higher infection risk, all exacerbating circulatory failure. Additionally, these patients may be less likely to receive advanced CS therapies due to multimorbidity and expected weaning difficulties with poorer long‐term outcome.

Suggesting that also a qualitative impact may disproportionately influence CS outcomes, underscoring the urgent need for further research to identify high‐risk subgroups and enable in‐depth phenotyping. This includes investigating specific clusters of comorbidities, as the combined occurrence of chronic conditions may confer synergistic risk beyond the sum of individual comorbidities. In the future, machine learning‐based clustering approaches could help to delineate such phenotypes, ideally aiming from the outset for simple clinical applicability.

### Comorbidity burden and treatment modalities

In this study, CS patients with a higher comorbidity burden were less likely to receive tMCS and mechanical ventilation, raising concerns about potential treatment disparities in this high‐risk population. Several factors may explain these findings. First, treating physicians may anticipate a limited potential for recovery, given the overall compromised baseline conditions resulting from a longstanding chronic burden and associated frailty. Comorbidity burden often serves as a surrogate for frailty status, which is increasingly recognized as a key factor influencing treatment decisions and outcomes.^32^ Second, the presence of multiple comorbidities could significantly alter the risk–benefit profile of advanced CS therapies, potentially increasing susceptibility to device‐related complications, such as restricted device access. In this analysis, a higher cumulative comorbidity burden was independently associated with a higher incidence of acute kidney injury. The incidence of acute kidney injury complicating CS is high, ranging from 20% to 35%, and is independently associated with worse clinical outcome.^33^, ^34^ Third, heart replacement therapies, including LVAD implantation and heart transplantation, have been associated with worse outcomes in patients with a higher comorbidity burden.^35^, ^36^ As a result, patients with high comorbidity burden may often be deemed ineligible for these definitive therapies, further limiting their treatment options. This underscores the importance of carefully evaluating the indication for tMCS to avoid futile interventions in cases where no long‐term therapeutic goal or recovery potential exists. Finaly, patients with a higher comorbidity burden may also have a lower likelihood of receiving guideline‐recommended medical therapies during hospitalization, either due to contraindications, or clinical inertia in the context of complex multimorbidity.

As comorbidity burden remains largely non‐modifiable throughout the CS trajectory, its strong association with clinical outcomes in this study underscores the need for early consideration in risk assessment. Integrating subtype‐specific characteristics of chronic conditions into prognostic evaluation is essential, particularly within the early CS phase in a shock team approach, to optimize clinical decision‐making. Risk prediction models as the COMRI‐CS score could support this approach, incorporating high‐risk comorbidities and a select set of readily available CS variables, demonstrating effective risk stratification. However, further in‐depth comorbidity phenotyping is needed to enhance its prognostic precision and clinical applicability.

### Limitations

This study is based on a non‐randomized dataset, which is a primary limitation as it precludes drawing causal conclusions. As sufficient data were only available for short‐term or in‐hospital outcomes, it remains uncertain whether these effects persist over longer‐time periods. Although all involved CSWG centres are large tertiary care centres with substantial experience in managing CS and implementing advanced CS therapies, this may have contributed to a higher utilization of tMCS within this cohort. Treatment decisions regarding vasoactive drugs and tMCS devices were likely influenced by provider preferences, potentially impacting outcomes.

Regarding specific limitations in assessing comorbidity burden, reliable data were only available for the pre‐defined comorbidities included in the dataset, potentially leading to an underestimation of their impact on outcomes. Additionally, no detailed classification of disease severity or stages within the respective comorbidities was performed.

## Conclusions

In this large, multicentre, real‐world cohort of CS patients, comorbidity burden was highly prevalent and associated with increased in‐hospital mortality, with a more pronounced impact in HF‐CS compared to AMI‐CS. Distinct high‐risk patterns were observed, highlighting the heterogeneity in their impact on clinical outcomes. As certain comorbidity burden may be non‐modifiable throughout the CS trajectory, these findings underscore the need to consider high‐risk subgroups in early risk assessment to optimize clinical decision‐making.

## Supporting information


**Appendix S1.** Supporting Information.
