# Supplementary material for: Impact of comorbidity burden on outcome in patients with cardiogenic shock: A Cardiogenic Shock Working Group analysis
Source: Eur J Heart Fail. 2025 Sep 16;27(11):2244–58. doi: 10.1002/ejhf.70017 (PMC12765368; doi:10.1002/ejhf.70017)
Supplement: Supplementary file 1 — Appendix S1. Supporting Information. [file EJHF-27-2244-s001.docx]

***SUPPLEMENTARY APPENDIX***

***Original Research Manuscript:***

***Impact of Comorbidity Burden on Outcome in Patients with Cardiogenic Shock***

Jonas Sundermeyer, MD; Song Li, MD; Van-Khue Ton, MD, PhD; Rachna Kataria, MD; Elric Zweck, MD; Kevin John, MD; Manreet K. Kanwar, MD; Jaime Hernandez-Montfort, MD, MPH; Shashank S. Sinha, MD, MSc; A. Reshad Garan, MD; Jacob Abraham, MD; Vanessa Blumer, MD; Ajar Kochar, MD; Karthikeyan Ranganathan, MD; Gavin W. Hickey MD; Mohit Pahuja, MD; Scott Lundgren, MD; Sandeep Nathan MD; Esther Vorovich, MD; Shelley Hall, MD; Wissam Khalife, MD; Andrew Schwartzman, MD; Ju Kim, MD; Oleg Alec Vishnevsky, MD; Justin Fried, MD; Mary Jane Farr, MD; Joseph Mishkin, MD; I-Hui Chang, MD; Onyedika Ilonze, MD; Alexandra Arias, MD; Jun Nakata, MD; Jeffrey Marbach, MD; Hiram Bezerra, MD; Ann Gage, MD; Joyce Wald, MD; Sunu Thomas, MD; Faisal Rahman, MD; Amirali Masoumi, MD; Aasim Afsal, MD; Salman Gohar, MD; Rachel Goodman, MD; Karol D. Walec, BS; Peter Natov, MD; ﻿Borui Li, MA; Paavni Sangal, MPH; ﻿Qiuyue Kong, MS; Peter Zazzali, MS, MPH; Neil M. Harwani, MS; Saraschandra Vallabhajosyula, MD; Arvind Bhimaraj, MD;Claudius Mahr, MD; Daniel Burkhoff, MD, PhD; and Navin K. Kapur, MD

***Supplementary Methods****:*

- ***Supplementary Methods 1:*** *COMRI-CS.*

***Supplementary Tables:***

- ***Supplementary Table 1****.* Characteristics stratified by the AMI-CS vs. HF-CS.
- ***Supplementary Table 2****.* Prevalence of multiple comorbidities in patients with HF-CS and AMI-CS.

***Supplementary Figures:***

- ***Supplementary Figure 1:*** Association between comorbidity burden and HF-CS vs. AMI-CS.
- ***Supplementary Figure 2:*** In-hospital outcome in the overall CSWG cohort. Supplementary
- ***Supplementary Figure 3***: Association between comorbidity burden and in-hospital mortality.
- ***Supplementary Figure 4***: Association between distinct comorbidities and in-hospital mortality, unadjusted analysis.
- ***Supplementary Figure 5:*** Association between distinct combinations of comorbidities and in-hospital mortality in all-cause cardiogenic shock.
- ***Supplementary Figure 6.*** Comorbidity burden and in-hospital complications.
- ***Supplementary Figure 7:*** Comorbidity burden and risk for in-hospital complications.
- ***Supplementary Figure 8:*** Impact of comorbidity burden on treatment utilization.
- ***Supplementary Figure 9:*** Group size, ROC curve and Bootstrap distribution in the derivation cohort.
- **Supplementary Figure 10:** Performance of COMRI-CS in the external validation cohort.

***Supplementary Methods 1:*** *COMRI-CS.*

To derive and internally/externally validate the Comorbidity Risk Index for Cardiogenic Shock (COMRI-CS), high-risk comorbidities were identified based on their association with in-hospital mortality using multivariable logistic regression analyses, as described before. Readily available parameters at admission, including age, sex, lactate, creatinine, and CS etiology (AMI-CS=yes), were incorporated into the model. The final predictive model was developed by transforming these variables into a clinically pragmatic, heuristic point-based scoring system. Point allocation was guided by the strength of association with mortality (based on odds ratios derived from a multivariable logistic regression including all score components and their respective subcategories), clinical plausibility, and findings from previous publications on predictive risk modeling in cardiogenic shock.

The derivation cohort included 4,355 patients with all-cause CS from the CSWG registry, further stratified into AMI-CS (n=1,255) and HF-CS (n=2,286). There were no missing data among the selected variables in this cohort. Receiver operating characteristic curves were calculated to assess the discriminatory performance of COMRI-CS (values ranging from 0.5 for no discrimination to 1.0 for best discrimination).

To assess the external validity and generalizability of COMRI-CS, the final predictive model was applied to an independent cohort from the MIMIC-IV database, a large-scale, publicly available repository of de-identified ICU patient data from Beth Israel Deaconess Medical Center (BIDMC), Boston, MA. The validation cohort included 3,188 patients with CS. The same inclusion and exclusion criteria, as well as identical statistical analyses, were applied to ensure comparability between the derivation and validation cohorts.

***Supplementary Tables***

***Supplementary Table 1****.* Characteristics stratified by the AMI-CS vs. HF-CS.

|  | **AMI-CS**  **(N=1,809)** | **HF-CS**  **(N=3,652)** | **P-value** |
| --- | --- | --- | --- |
| **Demographics** | | | |
| Male | 1,302 / 1,808 (72.0%) | 2,641 / 3,650 (72.4%) | 0.8 |
| Age | 65 (57, 73) | 61 (50, 68) | <0.001 |
| Asian | 119 / 1,809 (6.6%) | 101 / 3,652 (2.8%) | <0.001 |
| White | 1,248 / 1,809 (69%) | 2,265 / 3,652 (62%) | <0.001 |
| Other Race | 114 / 1,809 (6.3%) | 105 / 3,652 (2.9%) | <0.001 |
| Body Mass Index (kg/m²) | 27.7 (24.4, 32) | 28 (24, 32.7) | 0.8 |
| **Medical History** | | | |
| Hypertension | 1,173 / 1,786 (65.7%) | 2,365 / 3,642 (64.9%) | 0.6 |
| Diabetes mellitus | 796 / 1,793 (44.4%) | 1,502 / 3,647 (41.2%) | 0.024 |
| Atrial fibrillation/flutter | 211 / 1,784 (11.8%) | 1,524 / 3,648 (41.8%) | <0.001 |
| Chronic kidney disease | 314 / 1,786 (17.6%) | 1,368 / 3,649 (37.5%) | <0.001 |
| Peripheral vascular disease | 194 / 1,783 (10.9%) | 333 / 3,647 (9.1%) | 0.041 |
| COPD | 176 / 1,789 (9.8%) | 559 / 3,647 (15.3%) | <0.001 |
| Asthma | 86 / 1,693 (5.1%) | 331 / 3,545 (9.3%) | <0.001 |
| Liver disease | 38 / 1,710 (2.2%) | 209 / 3,579 (5.8%) | <0.001 |
| Anemia | 197 / 1,691 (11.6%) | 753 / 3,545 (21.2%) | <0.001 |
| History of stroke/TIA | 182 / 1,789 (10.2%) | 525 / 3,645 (14.4%) | <0.001 |
| Severe valve disease | 149 / 1,789 (8.3%) | 905 / 3,644 (24.8%) | <0.001 |
| Prior CAD | 715 / 1,651 (43.3%) | 1,537 / 3,533 (43.5%) | 0.9 |
| History of HF | 477 / 1,780 (26.8%) | 2,945 / 3,647 (80.8%) | <0.001 |
| History of MI | 425 / 1,784 (23.8%) | 818 / 3,647 (22.4%) | 0.3 |
| Number of comorbidities | 2 (1, 4) | 4 (2, 6) | <0.001 |
| **Laboratory Values Admission** | | | |
| Lactate (mEq/L) | 2.6 (1.5, 5.6) | 2 (1.4, 3.4) | <0.001 |
| pH | 7.3 (7.2, 7.4) | 7.4 (7.3, 7.4) | <0.001 |
| ALT (IU/L) | 50.5 (25, 100) | 30 (18, 60) | <0.001 |
| AST (IU/L) | 85 (37, 196) | 36 (24, 70) | <0.001 |
| Serum Creatinine (mg/dL) | 1.3 (1, 1.8) | 1.5 (1.1, 2) | <0.001 |
| Blood Urea Nitrogen (mg/dL) | 23.3 (17, 37) | 30 (21, 44) | <0.001 |
| Sodium (mEq/L) | 137 (134, 140) | 136 (133, 139) | <0.001 |
| Potassium (mEq/L) | 4.1 (3.7, 4.6) | 4.1 (3.7, 4.6) | 0.8 |
| HCO3 (mEq/L) | 21 (18, 24) | 23 (20, 26) | <0.001 |
| Total Bilirubin (mg/dL) | 0.7 (0.4, 1.1) | 1.1 (0.7, 1.8) | <0.001 |
| Hemoglobin (g/dL) | 12.4 (10.3, 14.1) | 12.4 (10.6, 14) | 0.4 |
| **Echocardiography baseline** | | | |
| LVEF baseline | 27 (20, 38) | 19.5 (13, 27) | <0.001 |
| **Hemodynamics** | | | |
| SBP | 112 (97, 132) | 108 (96, 122) | <0.001 |
| DBP | 71 (60, 83) | 71 (62, 81) | 0.2 |
| Heart Rate (bpm) | 90 (76, 106) | 93 (79, 108) | <0.001 |
| MAP | 83 (73, 95) | 83 (74, 92.6) | 0.8 |
| Central Venous Pressure | 11 (7, 15) | 13 (8, 18) | 0.018 |
| Right Atrial Pressure | 12 (9, 16) | 13 (9, 17) | 0.12 |
| Pulmonary Artery Systolic Pressure | 39 (31, 48) | 46 (36, 57) | <0.001 |
| Pulmonary Artery Diastolic Pressure | 22 (17, 27) | 25 (19, 31) | <0.001 |
| Pulmonary Capillary Wedge Pressure | 21 (16, 27) | 23 (17, 28) | 0.4 |
| Cardiac Output | 4.1 (3.1, 5.2) | 3.9 (3.2, 4.8) | 0.2 |
| **SCAI Shock Stage** | | | |
| SCAI B baseline | 215 / 1,809 (11.9%) | 591 / 3,652 (16.2%) | <0.001 |
| SCAI C baseline | 274 / 1,809 (15.1%) | 668 / 3,652 (18.3%) | 0.004 |
| SCAI D baseline | 329 / 1,809 (18.2%) | 447 / 3,652 (12.2%) | <0.001 |
| SCAI E baseline | 435 / 1,809 (24%) | 335 / 3,652 (9.2%) | <0.001 |
| **Mechanical circulatory support** | | | |
| ECMO (VA) | 348 / 1,809 (19.2%) | 403 / 3,652 (11%) | <0.001 |
| Impella CP | 352 / 1,809 (19.5%) | 169 / 3,652 (4.6%) | <0.001 |
| Impella 2.5 | 2 / 1,809 (0.1%) | 2 / 3,652 (0.1%) | 0.6 |
| Impella 5.0 | 5 / 1,809 (0.3%) | 13 / 3,652 (0.4%) | 0.6 |
| Impella 5.5 | 225 / 1,809 (12.4%) | 593 / 3,652 (16.2%) | <0.001 |
| IABP | 430 / 1,809 (23.8%) | 845 / 3,652 (23.1%) | 0.6 |
| **Other Treatments** | | | |
| Number of vasoactive drugs max | |  |  |
| 0 | 68 / 965 (7.0%) | 104 / 1,978 (5.3%) |  |
| 1 | 260 / 965 (26.9%) | 764 / 1,978 (38.6%) |  |
| 2 | 264 / 965.0 (27.4%) | 510 / 1,978 (25.8%) |  |
| 3 | 220.0 / 965 (22.8%) | 353 / 1,978 (17.8%) |  |
| 4 | 120 / 965.0 (12.4%) | 192 / 1,978 (9.7%) |  |
| 5 | 29.0 / 965 (3.0%) | 43 / 1,978 (2.2%) |  |
| Mechanical ventilation | 1,322 / 1,798 (73.5%) | 2,030 / 3,628 (56.0%) | <0.001 |
| RRT | 411 / 1,803 (22.8%) | 779 / 3,636 (21.4%) | 0.2 |
| **Outcome** | | | |
| Heart Transplant | 34 / 1,809 (1.9%) | 511 / 3,649 (14%) | <0.001 |
| LVAD Implantation | 184 / 1,801 (10.2%) | 590 / 3,634 (16.2%) | <0.001 |
| In-Hospital Mortality | 683 / 1,809 (37.8%) | 841 / 3,652 (23%) | <0.001 |
| **In-hospital complications** | | | |
| In-Hospital PCI | 359 / 1,020 (35.2%) | 127 / 3,542 (3.6%) | <0.001 |
| In-Hospital CABG | 138 / 1,706 (8.1%) | 78 / 3,547 (2.2%) | <0.001 |
| In-Hospital Cardiac Arrest | 579 / 1,781 (32.5%) | 422 / 3,631 (11.6%) | <0.001 |
| Limb Ischemia | 146 / 1,777 (8.2%) | 107 / 3,626 (3%) | <0.001 |
| Acute Kidney Injury | 949 / 1,730 (54.9%) | 2,142 / 3,582 (59.8%) | <0.001 |
| Bleeding Requiring Surgery | 86 / 1,799 (4.8%) | 122 / 3,641 (3.4%) | 0.010 |
| Bleeding Requiring Transfusion | 497 / 1,778 (28%) | 569 / 3,609 (15.8%) | <0.001 |
| Hemolysis | 191 / 1,766 (10.8%) | 272 / 3,598 (7.6%) | <0.001 |

Binary variables are presented as absolute numbers and relative frequencies, and comparisons were conducted using Pearson’s Chi-squared test or Fisher's exact test. Continuous variables are shown as the median with interquartile range (IQR) and analyzed using the Kruskal-Wallis test. AMI-CS = acute myocardial infarction–cardiogenic shock; ALT = alanine aminotransferase; AST = aspartate aminotransferase; CAD = coronary artery disease; COPD = chronic obstructive pulmonary disease; HCO3 = sodium bicarbonate; HF = heart failure; HF-CS = heart failure–cardiogenic shock; LVEF = left ventricular ejection fraction; MAP = mean arterial pressure; MI = myocardial infarction; RRT = renal replacement therapy; SBP = systolic blood pressure; SCAI = society for cardiovascular angiography and interventions; TIA = transient ischemic attack; VA-ECMO = veno-arterial extracorporeal membrane oxygenation.

***Supplementary Table 2****.* Prevalence of multiple comorbidities in patients with HF-CS and AMI-CS.

|  | **All-Cause N (%)** | **HF-CS N (%)** | **AMI-CS N (%)** |
| --- | --- | --- | --- |
| ≥1 Comorbidities | 6087 (89.3%) | 3426 (93.8%) | 1499 (82.9%) |
| ≥2 Comorbidities | 5309 (77.9%) | 3138 (85.9%) | 1194 (66%) |
| ≥3 Comorbidities | 4390 (64.4%) | 2735 (74.9%) | 865 (47.8%) |
| ≥4 Comorbidities | 3442 (50.5%) | 2243 (61.4%) | 604 (33.4%) |
| ≥5 Comorbidities | 2527 (37.1%) | 1702 (46.6%) | 407 (22.5%) |
| ≥6 Comorbidities | 1664 (24.4%) | 1141 (31.2%) | 269 (14.9%) |
| ≥7 Comorbidities | 986 (14.5%) | 688 (18.8%) | 157 (8.7%) |

AMI-CS = acute myocardial infarction–cardiogenic shock; HF-CS = heart failure–cardiogenic shock.

***Supplementary Figures:***

***Supplementary Figure 1:*** Association between comorbidity burden and HF-CS vs. AMI-CS.

***
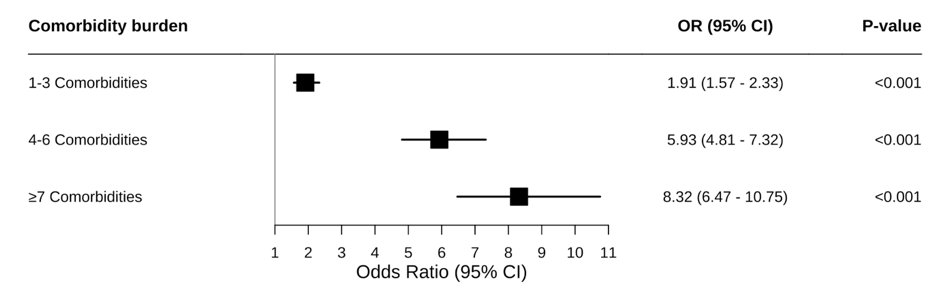
***

A higher comorbidity burden was significantly associated with HF-CS (vs. AMI-CS), adjusted for age and sex.


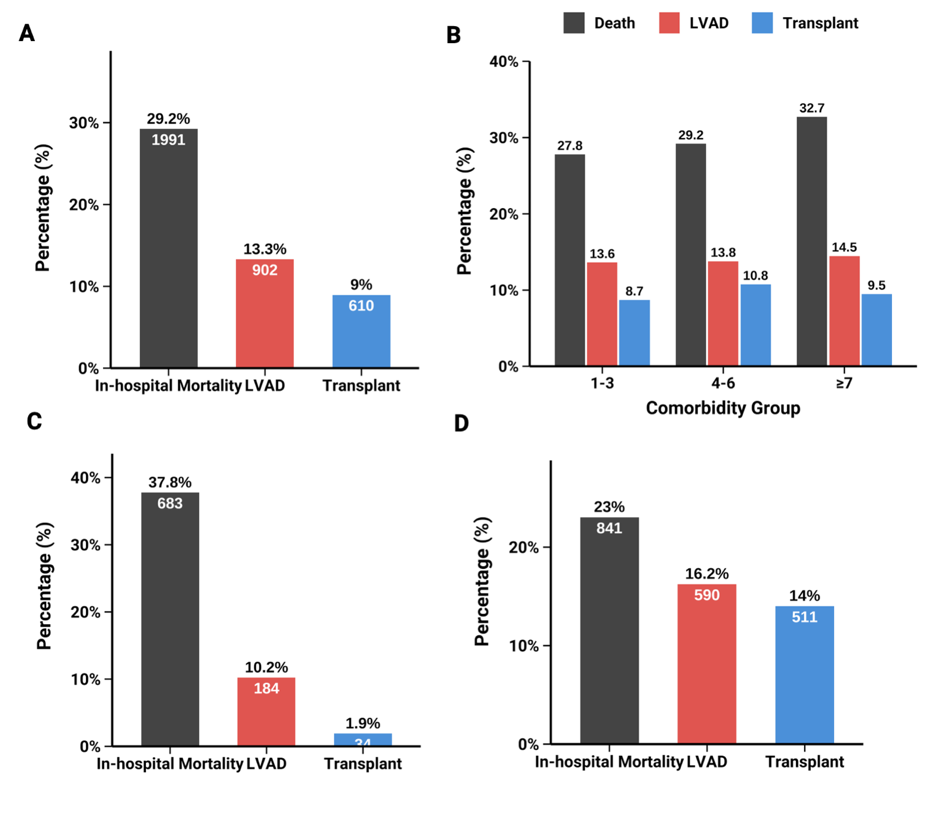
***Supplementary Figure 2:*** In-hospital outcome in the CSWG cohort.

Crude clinical outcome in all-cause CS (**A**) and stratified by comorbidity burden (**B**). Crude clinical outcome in AMI-CS (**C**), and HF-CS (**D**). AMI-CS = acute myocardial infarction-related cardiogenic shock, HF-CS = heart failure-related cardiogenic shock, LVAD = left ventricular assist device.

***Supplementary Figure 3:*** Association between comorbidity burden and in-hospital mortality.

***
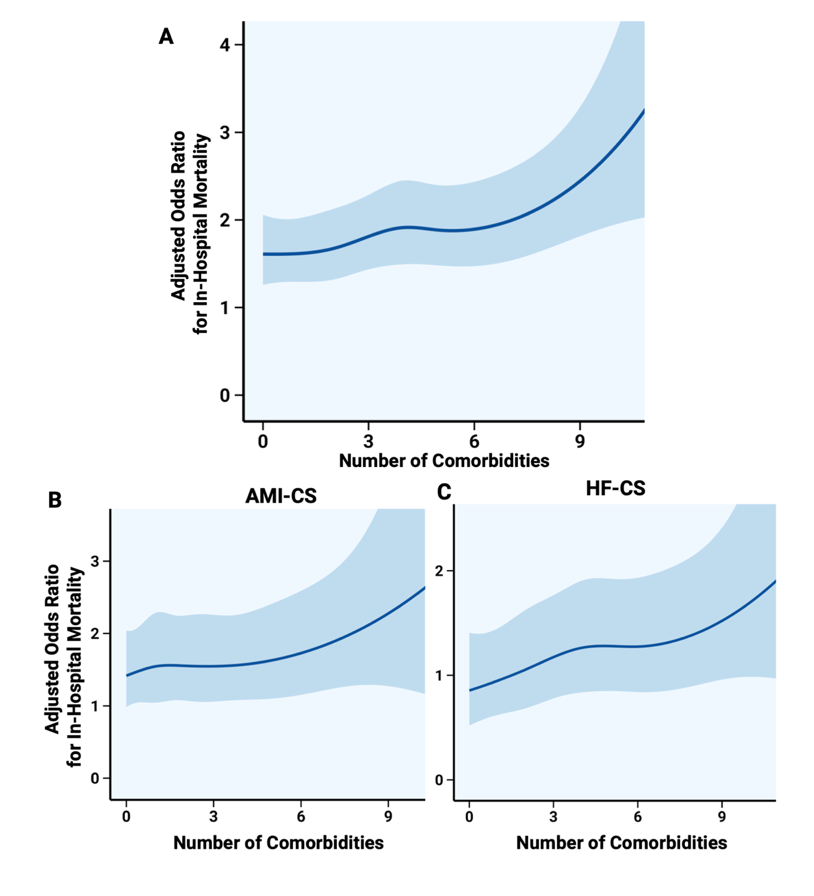
***

To assess the potential non-linear associations between multimorbidity burden and in-hospital mortality, multivariable logistic regression models were applied, modeling the number of comorbidities as a continuous variable using restricted cubic splines. All-cause CS (**A**), AMI-CS (**B**) and HF-CS (**C**). Adjusted for age, sex, lactate, creatinine, out-of-hospital cardiac arrest. AMI-CS = acute myocardial infarction-related cardiogenic shock, HF-CS = heart failure-related cardiogenic shock.

***Supplementary Figure 4:*** Association between distinct comorbidities and in-hospital mortality, unadjusted analysis.


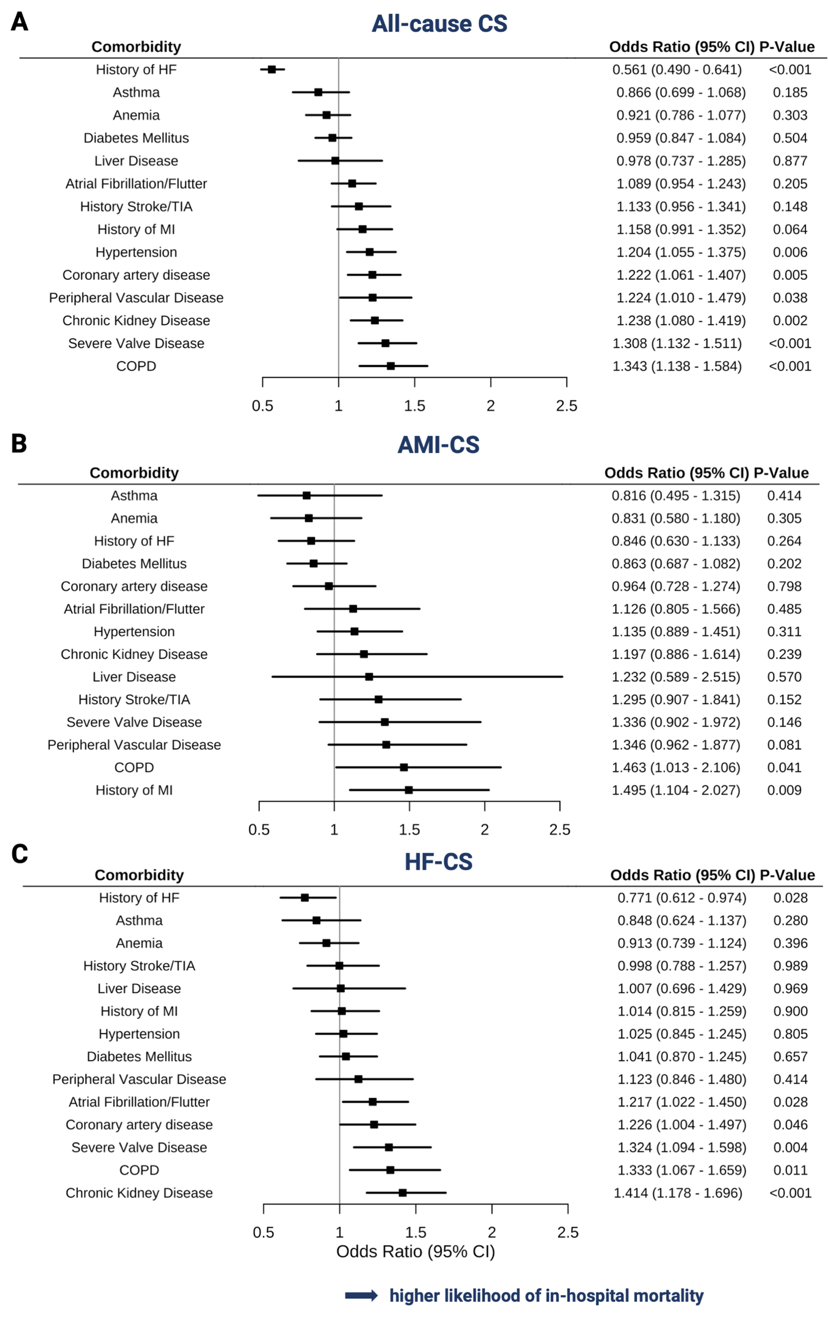


AMI-CS = acute myocardial infarction-related cardiogenic shock, HF-CS = heart failure-related cardiogenic shock.

***Supplementary Figure 5:*** Association between distinct combinations of comorbidities and in-hospital mortality in all-cause cardiogenic shock.


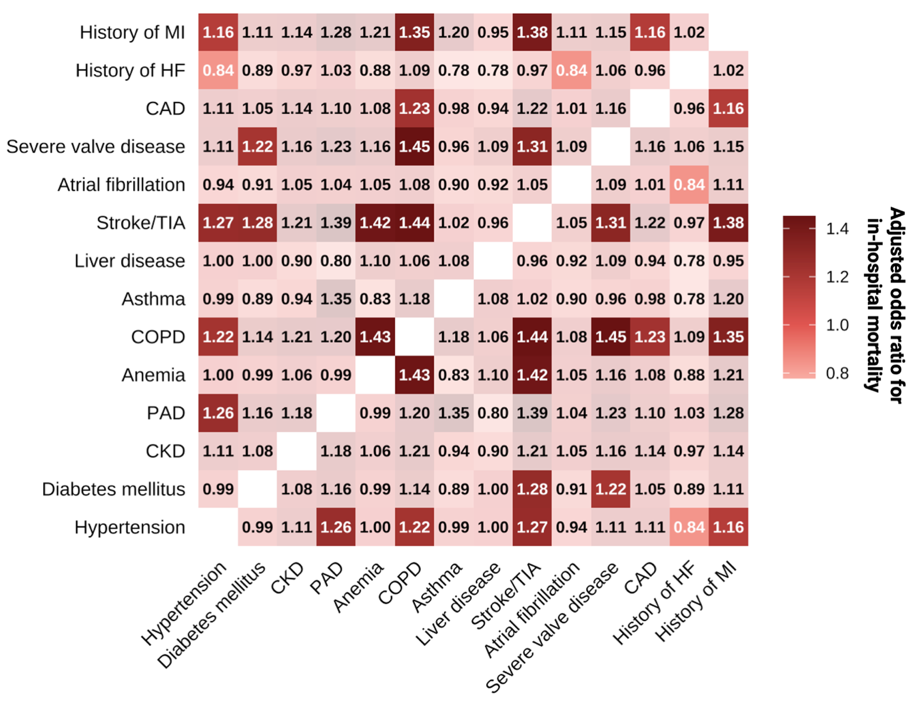


Association between distinct comorbidity combinations and in-hospital mortality, calculated using multivariable logistic regression models, adjusted for age and sex. Statistically significant results (p<0.05) are highlighted in color.

***Supplementary Figure 6.*** Comorbidity burden and in-hospital complications.


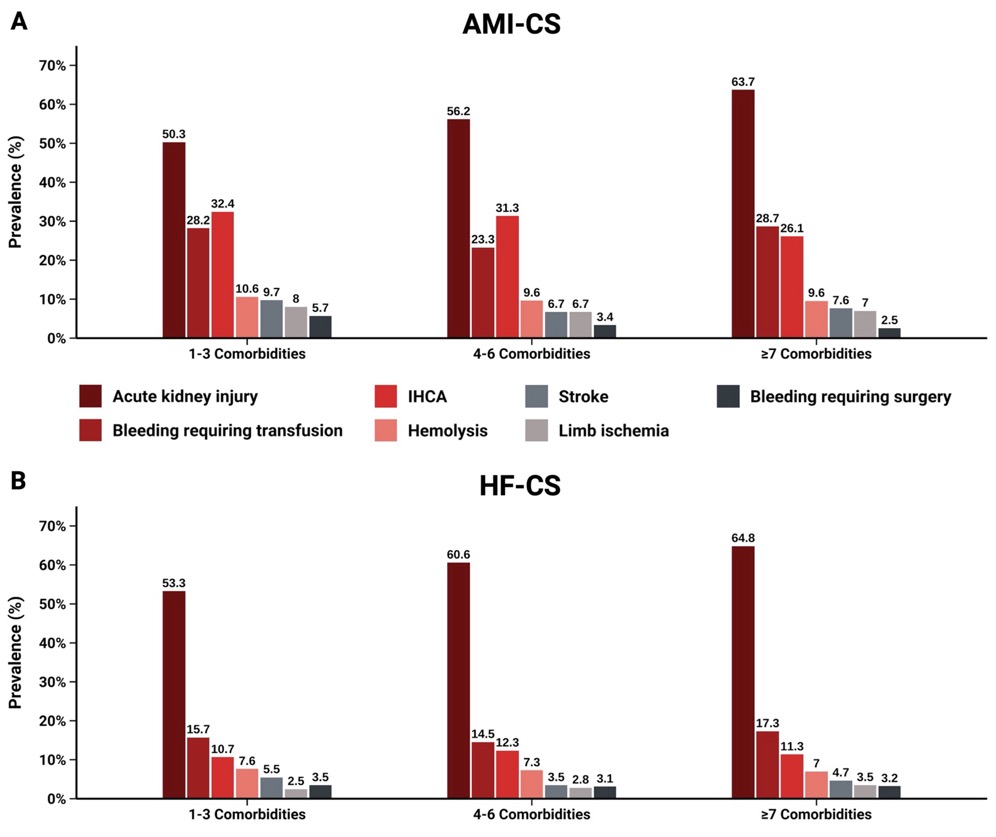


Prevalence of in-hospital complications stratified by comorbidity burden in AMI-CS (**A**) and HF-CS (**B**). A higher comorbidity burden was associated with an increased risk of acute kidney injury in both CS subtypes. AMI-CS = acute myocardial infarction-related cardiogenic shock; HF-CS = heart failure-related cardiogenic shock; IHCA = intra-hospital cardiac arrest.

***Supplementary Figure 7:*** Comorbidity burden and risk for in-hospital complications.


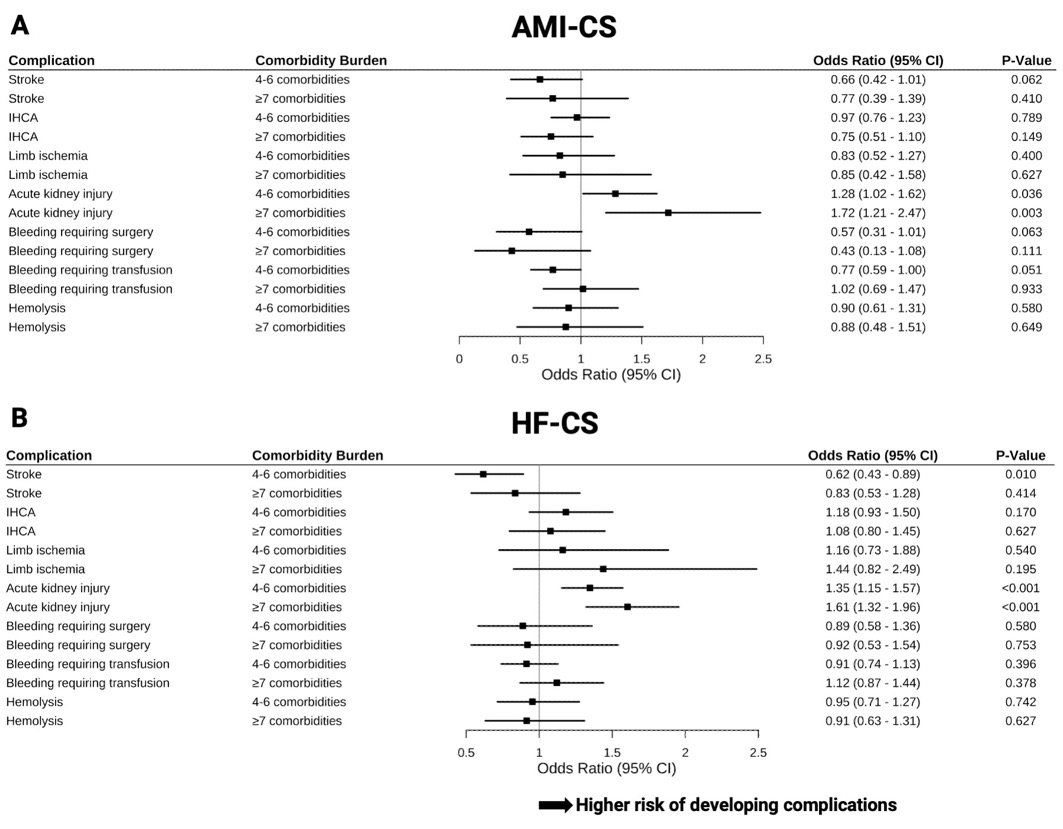


Association between higher comorbidity burden (4-6 or ≥7 comorbidities vs. 1-3 comorbidities) and risk for in-hospital complications in AMI-CS (**A**) and HF-CS (**B**).

***Supplementary Figure 8:*** Impact of comorbidity burden on treatment utilization.


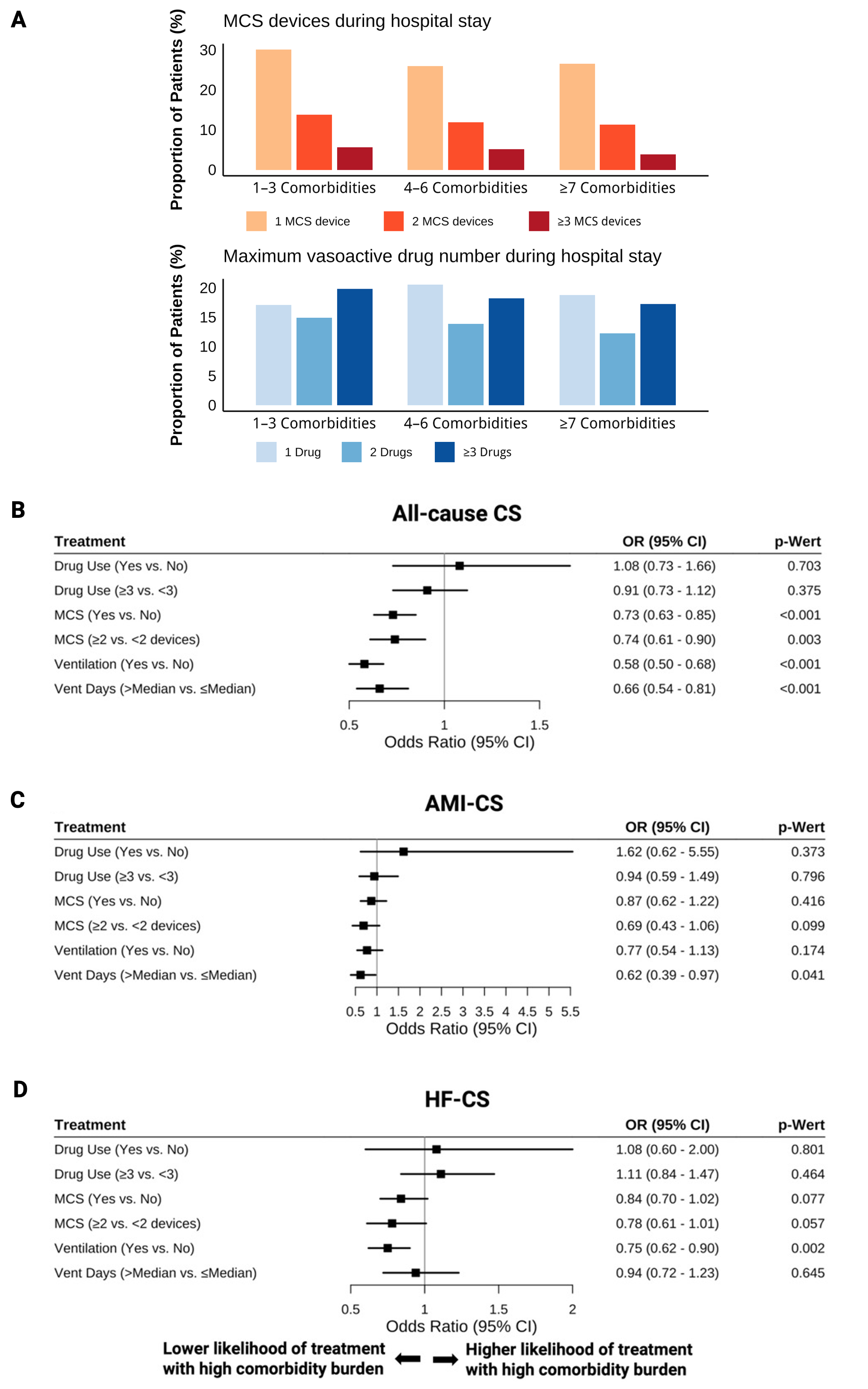


(**A**) Treatment utilization across comorbidity burden. Maximum number of MCS devices during hospitalization, counting all devices placed (including sequential use after interim removal); maximum number of concurrently administered vasoactive agents during the hospital stay. (**B**) Impact of high comorbidity burden (≥7 comorbidities vs. 1-3 comorbidities) on treatment utilization in all-cause CS, (**C**) AMI-CS, and (**D**) HF-CS. AMI-CS = acute myocardial infarction-related cardiogenic shock; HF-CS = heart failure-related cardiogenic shock; MCS = mechanical circulatory support.

***Supplementary Figure 9:*** Group size, ROC curve and Bootstrap distribution in the derivation cohort.


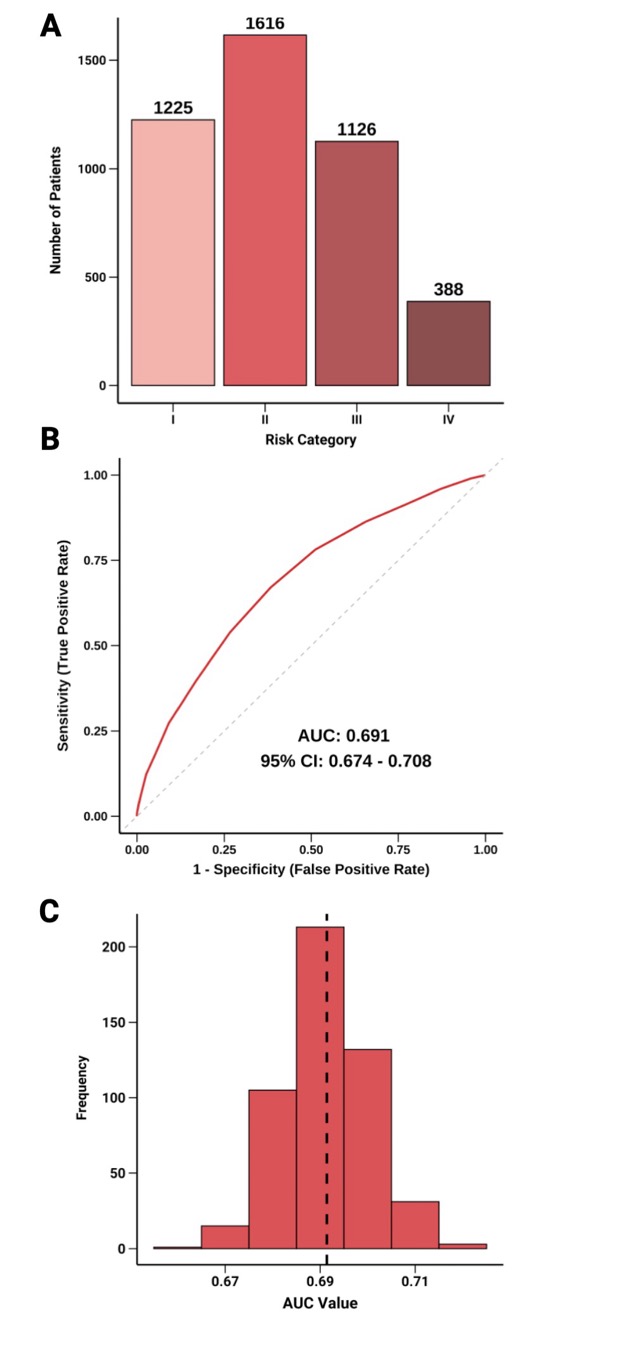


Number of patients across risk categories (**A**), ROC curve (**B**) and bootstrap distribution (**C**) in the derivation cohort. AUC = Area under the curve.

***Supplementary Figure 10:*** Performance of COMRI-CS in the external validation cohort.


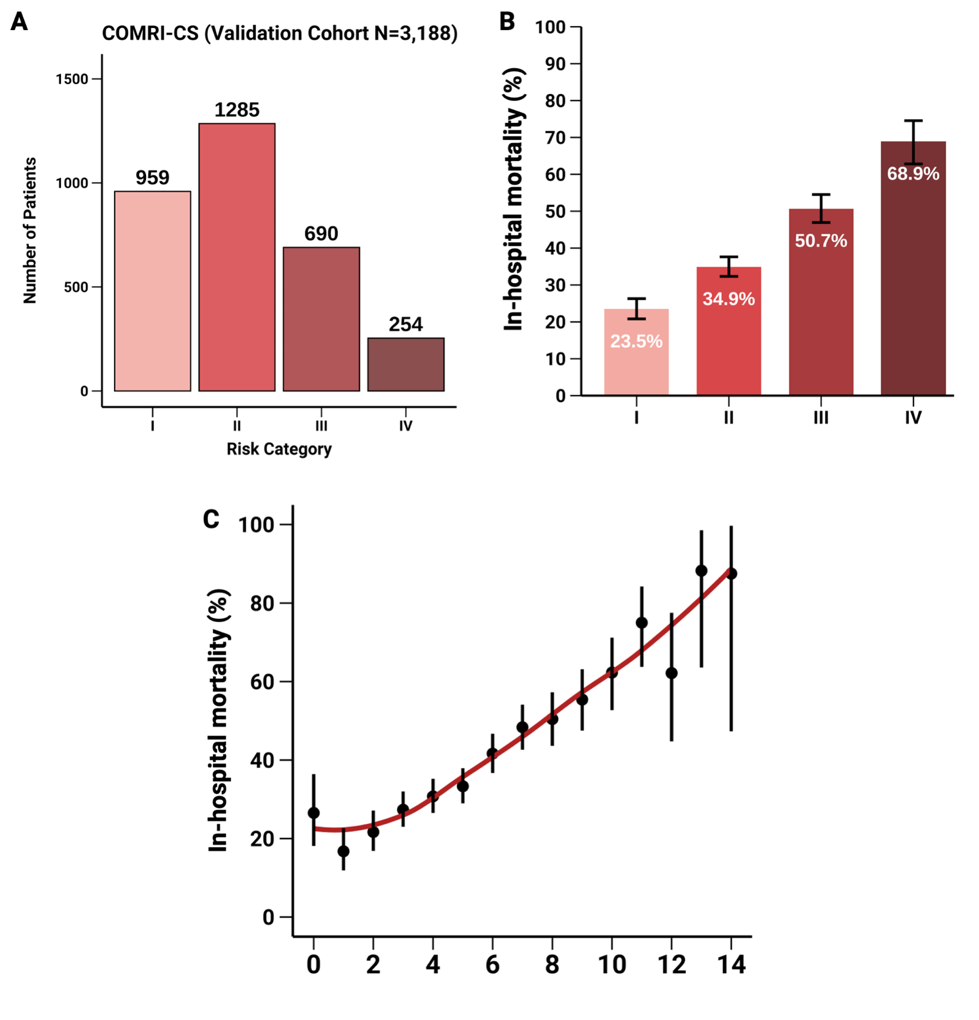


Number of patients of the external validation cohort across risk categories (**A**). In-hospital mortality rates within the risk classes for all-cause CS in the external validation cohort (**B**). In-hospital mortality rates across the COMRI-CS score spectrum in the external validation cohort (**C**). Bars represent 95% confidence intervals.
